# The RoboCOS Study: Development of an international core outcome set for the comprehensive evaluation of patient, surgeon, organisational and population level impacts of robotic assisted surgery

**DOI:** 10.1371/journal.pone.0283000

**Published:** 2023-03-30

**Authors:** Clare Robertson, Shafaque Shaikh, Jemma Hudson, Patrick Garfjeld Roberts, David Beard, Terry Mackie, Cameron Matthew, Craig Ramsay, Katie Gillies, Marion Campbell

**Affiliations:** 1 Health Services Research Unit, University of Aberdeen, Aberdeen, United Kingdom; 2 Aberdeen Royal Infirmary, NHS Grampian, Aberdeen, United Kingdom; 3 Nuffield Department of Orthopaedics, Surgical Interventions Trials Unit, Rheumatology & Musculoskeletal Sciences, University of Oxford, Oxford, United Kingdom; 4 Public Partner, Aberdeen, United Kingdom; Universitat Luzern, SWITZERLAND

## Abstract

**Background:**

The introduction of robot-assisted surgery is costly and requires whole system transformation, which makes the assessment of benefits (or drawbacks) complex. To date, there has been little agreement on which outcomes should be used in this regard. The aim of the RoboCOS study was to develop a core outcome set for the evaluation of robot-assisted surgery that would account for its impact on the whole system.

**Methods:**

Identification of a long-list of potentially relevant outcomes through systematic review of trials and health technology assessments; interviews with individuals from a range of stakeholder groups (surgeons, service managers, policy makers and evaluators) and a focus group with patients and public; prioritisation of outcomes via a 2-round online international Delphi survey; consensus meeting.

**Results:**

721 outcomes were extracted from the systematic reviews, interviews and focus group which were conceptualised into 83 different outcome domains across four distinct levels (patient, surgeon, organisation and population) for inclusion in the international Delphi prioritisation survey (128 completed both rounds). The consensus meeting led to the agreement of a 10-item core outcome set including outcomes at: patient level (treatment effectiveness; overall quality of life; disease-specific quality of life; complications (including mortality); surgeon level (precision/accuracy; visualisation); organisation (equipment failure; standardisation of operative quality; cost-effectiveness); and population (equity of access).

**Conclusion:**

The RoboCOS core outcome set, which includes the outcomes of importance to all stakeholders, is recommended for use in all future evaluations of robot-assisted surgery to ensure relevant and comparable reporting of outcomes.

## Introduction

The uptake of innovative minimally invasive technology, particularly robot-assisted surgery (RAS), is expanding exponentially. Procedures such as robotic prostatectomy were adopted early with this technology, and technology specifications have now developed to allow wider inclusivity of patient and disease groups like colorectal cancer, gynaecological cancer and lung cancer. The Royal College of Surgeons, England [1] commissioned report predicts the rapid expansion of RAS across the UK and internationally. RAS has been under constant evolution with the next 3–5 years expected to involve further significant developments including the introduction of novel artificial intelligence augmented RAS platforms and a consequent widening range of clinical application.

Unlike other surgical procedures, RAS requires whole system transformation involving reconfiguration of space for large physical kit, new training for surgical teams, novel ways of working and the alteration of clinical pathways [2,3]. As such, RAS comes with a new set of implementation challenges involving exponentially higher investments into infrastructure, alongside the acquisition and running costs of the technology. A minimum case volume is also generally required to achieve financial sustainability [4].

These complex and costly service transformation issues make the assessment of the net benefit (or drawbacks) of RAS complex and difficult. Any shortcomings or restrictions need to be measured against possible patient, clinician or system-related advantages of RAS, such as increased precision of surgery, or improved learning curve. The problem is compounded by the plethora and heterogeneity of outcomes currently used in clinical evaluations of RAS making comparison across studies difficult [5]. Given that RAS technology is highly likely to increase, there is an urgent need for healthcare organisations and other stakeholders to be able to make informed judgements of the overall “value” of RAS.

In other fields, the development of “core outcome sets” (COS) have been helpful in ensuring that the appropriate outcomes inform robust evaluation by decision makers [6]. The fundamental principle underpinning a COS is the agreement of a minimum set of outcomes of paramount importance to all key stakeholder groups (e.g. clinicians, patients, service managers, policy makers) that should be measured in all evaluations. This allows increased comparability across studies and also enables a comprehensive quantification of any impacts from a much wider perspective i.e. all relevant key stakeholders.

No Core Outcome Set exists for RAS and, whilst some areas of other clinical core outcome sets may have similarities and lend components, few have focused on the assessment of the whole-system change required by RAS with impacts at patient, surgeon, organisation and population levels. Our research, the Robotic Core Outcome Set (RoboCOS) study, aimed to address this gap–to develop a COS for the evaluation of RAS which would allow a fair and transparent mechanism for healthcare organisations and others to effectively assess the added value of RAS.

## Methods

**Fig 1:**
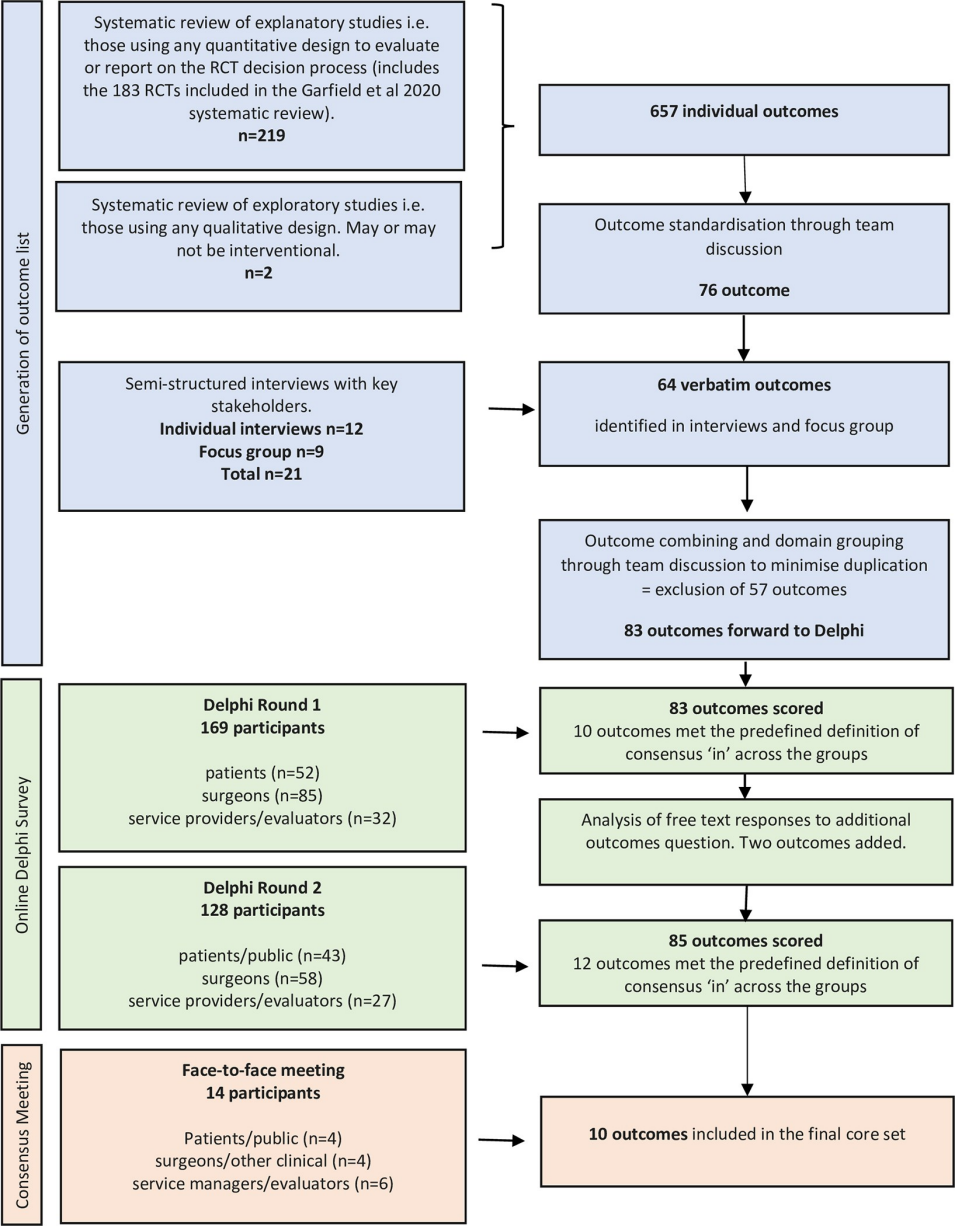
Core outcome set development overview.

The development of the core outcome set followed international COS-STAD (Core Outcome Set–Standards for Development) best practice guidelines for core outcome set development [7]. This involved three primary phases: 1) identification of all potential relevant outcomes; 2) conduct of an online Delphi survey to assess the relative importance of the different outcomes to a range of stakeholders and 3) the conduct of a consensus meeting to finalise the COS. The methods for each stage are outlined in Fig 1. The COS was pre-registered on the COMET database (https://www.comet-initiative.org/Studies/Details/1608).

### Development of the long-list of relevant outcomes

#### Systematic identification of outcomes from previously published literature

In order to identify what outcomes had been measured and reported in previous studies, we extracted all outcomes previously reported from three key sources.

- A recent systematic review of all previously published RAS randomised controlled trials [5]. As part of the conduct of that review, the investigators had extracted all outcomes used in the trials and this database of outcomes was made available for this study.
- The National Institute for Health Research/National Institute for Health and Care Excellence (NICE) evidence synthesis and technology assessment report on robot-assisted surgery [4].
- International policy-level health technology assessment evaluations of RAS–a systematic search of all available international health technology assessments for RAS was undertaken using the International Network of Agencies for Health Technology Assessment (INAHTA) database (search strategy available from supplementary file S1 in S1 File) [8].

Documents identified from those sources were supplemented through reference chaining.

A standardised data extraction form was developed and all recorded outcomes reported, the outcome definitions, and the measurement tools used. Data were also extracted on: study design; countries and institutions where data collected; robotic platforms used, safety considerations, number of participants; definitions of all included outcomes; time points of measurement for all outcomes; measurement tool used, diffusion and uptake of robotics, training and learning curves, health economic analyses, and whether patients or public were involved. Where Patient Reported Outcome Measures (PROMs) were included, all separate domains included in the PROMs were coded. For studies using qualitative methods, outcomes were extracted verbatim. Data extraction was undertaken by the lead researcher (CRo) with a random 10% dual data extraction conducted independently by KG and MC. Any disagreements were arbitrated by a third member of the team.

#### Interviews with stakeholders & focus group

In addition to identifying the outcomes that had already been cited in the literature, we also undertook interviews with key stakeholder groups to assess whether any important outcomes had been overlooked or excluded.

We sought to interview approximately 10 semi-structured interviews. A convenience sample of each stakeholder group (surgeons, service providers, evaluators) was identified by the project team to ensure a diverse range of experiences are captured including across geographical areas. The size of the sample was in line with numbers reported for the interview stage of consensus-based studies. Prospective participants were provided with an invitation letter and study information. Before the interview commenced the researcher obtained informed verbal consent from participants. The interviews were conducted via Microsoft Teams (Microsoft Teams (Microsoft Corporation, One Microsoft Way, Redmond, Washington). All interviews were audio-recorded and transcribed by a validated transcription service. Any outcomes identified were extracted together with the reasons used to justify their importance.

A focus group with patients and public partners was also convened. The analysis of the focus group was conducted in a similar manner to the interviews above. The topic guides for the interviews and focus group are presented in supplementary files S2 and S3 in S1 File.

All the specific outcomes identified via the literature review, the interviews and the focus groups were reviewed by the project team and classified into conceptually distinct outcome domains (see supplementary file S4 in S1 File for details of the outcome reduction).

### Online Delphi survey

To identify the relative importance of outcomes across stakeholder groups a 2-round Delphi consensus survey was conducted. Each outcome domain was listed together with a plain language definition on the online DelphiManager software [9]. The outcomes were listed individually but also grouped into relevant levels (e.g. patient level, surgeon level, organisation level and population level) to reflect the different types of outcomes identified (see supplementary file S5 in S1 File for details).

Stakeholder groups (surgeons, patients & public, service providers) were invited to participate in the Delphi survey through email distribution lists, known professional networks, social media and via direct recommendations to the research team (see supplementary file S6 in S1 File for details of organisations that circulated the survey). Explicit consent was not sought for the Delphi survey; instead, consent was implicit by completion and return of the questionnaire. There is no standard method for determining sample size calculations for Delphi studies; however, we set the minimum sample size required for analysis to be 10 participants per stakeholder group in line with a recent study by Harman *et al* [10].

Two rounds of scoring were conducted with feedback provided (their individual score and a summary distribution of scores by stakeholder group) between rounds. Respondents were asked to consider how important they thought each listed item was for the assessment for RAS services. Participants were asked to score each of the listed items using the Grading of Recommendations, Assessment, Development and Evaluations (GRADE) scale of 1 to 9 with a score of 1 to 3 being interpreted as having ‘limited importance’, 4 to 6 as ‘important but not critical’ and 7 to 9 as ‘critical’ [11]. Round 1 participants were also invited to note any additional outcomes–these were considered by the project team for inclusion in Round 2. No outcomes were removed between rounds. Only those who had completed scores on more that 75% of the outcomes in Round 1 were invited to complete Round 2.

### Consensus definitions

Following Round 2, the proportion of respondents scoring 1–3, 4–6, and 7–9 was calculated for each outcome. Each outcome was then classified as: indicative ‘consensus in’ (i.e. consensus that the outcome should be included in a core set), indicative ‘consensus out’ (i.e. consensus that the outcome should not be included in a core set) or indicative ‘no consensus’ (i.e. items that are equivocal and require further research for clarification). The original definition of consensus, based on previous COS studies, required that 70% or more of the entire group agreed the outcome was important (or not) and less than 15% scored it as not important [12,13].

However, so as to ensure at least one outcome from each of the 4 core areas was included in the final core set (as per OMERACT recommendations) [14] the thresholds for consensus were amended—but blinded to the outcomes. The revised criteria are presented in Table 1:

**Table 1:** Thresholds for achieving consensus ‘in’, consensus ‘out’ and no consensus.

	Patient outcomes	Other outcomes
**Consensus in**	>90% 7–9	>70% 7–9
**Consensus Out**	<70% 7–9	<50% 7–9
**No consensus**	70%-90% 7–9	50%-70% 7–9

### Consensus meeting

A virtual consensus meeting was held using Zoom (Zoom Video Communications, Inc., San Jose, California, U.S.)—the main aim being to agree the final core outcome set and provide an opportunity for discussion. Participants who had completed both rounds of the Delphi survey were invited to express an interest in attending the consensus meeting. Additional invitations were sent through social media and known professional networks to target patients and researchers.

Participants were sent a summary of the study in advance and a consensus matrix detailing the Round 2 summary scores for all outcomes–see supplementary information file S7 in S1 File. The consensus meeting was chaired by a trials methodologist with expertise in COS development methodology and consensus facilitation (MC). The consensus meeting was audio-recorded to capture the key points of the discussion.

Full details of the consensus meeting methods are provided in supplementary files S8 and S9 in S1 File. Briefly, outcomes that had reached indicative consensus ‘in’ across the whole group were presented first, followed by outcomes that reached indicative consensus ‘out’ across the whole group. Participants were asked to confirm they agreed (or not) with the inclusion or exclusion of these outcomes in the COS. Next, outcomes scored as ‘no consensus’ were presented in two groups: a) 12 outcomes that scored above 75% importance for patient outcomes and above 60% for other outcomes which were kept for discussion; and b) 19 outcomes that scored below 75% importance for patient outcomes and below 60% for other outcomes were proposed to be consensus out. A further four outcomes determined as consensus-out were also raised for discussion due to wide heterogeneity in scoring between the stakeholder groups (over 40% between the highest and lowest group ranking AND one group scored over 90% in the 7–9 score category).

Views for and against inclusion of the outcomes for which there was ‘no consensus’ were sought by the meeting chair with the expectation of adding additional outcomes to the core set “by exception”. Participants were further asked to consider whether the additional outcome was potentially subsumed within an outcome already in the core set. Following discussion, participants were invited to vote on each outcome anonymously using the polling function within Zoom–voting “yes” or “no” to the addition of the outcome to the core set. At least 70% of the participants had to vote in favour to pass the threshold for inclusion.

Following the assessment of all outcomes the final core set was reviewed again for possible duplication or repetition. Any outcomes felt to be duplications were then voted on using the same process as above (70% of the group had to agree to its removal).

### Research ethics

The protocol for the study was submitted to the study sponsor for initial review and classified as “service evaluation” and deemed not to require formal ethics approval by the joint College Ethics Review Board (CERB) for University of Aberdeen and NHS Grampian.

## Results

### Literature search

**Fig 2:**
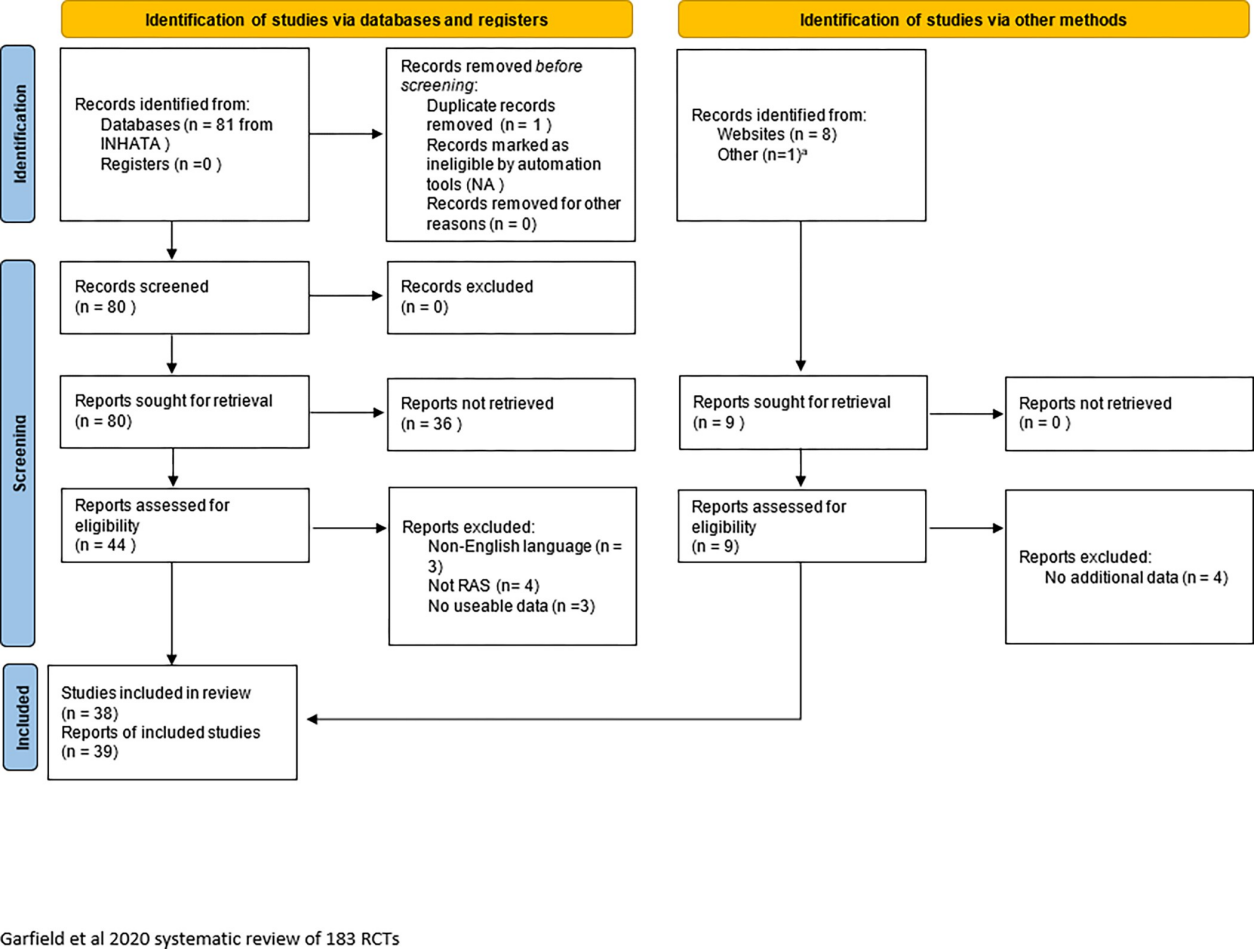
PRISMA flow diagram for identification of included studies in the RoboCOS systematic review.

In addition to the Garfjeld-Roberts *et al* [5] systematic review, the literature search identified 81 titles and abstracts for screening from the INAHTA database. A further eight reports were identified by hand-searching and known literature. A total of 53 full text reports were screened for eligibility, and 39 reports (from 38 studies) were included in the final analysis (see Fig 2 for the PRISMA flow diagram). The majority of studies identified from the literature search were health technology assessment reports of the clinical and cost-effectiveness of RAS, and two were qualitative studies using a realist evaluation method [15,16]. Of the studies identified in the additional literature search: 12 reports (from 11 studies) were conducted in Canada, 8 were from the UK, 4 were from the USA, 4 were from South Korea, 2 each were conducted from Australia, Australia and New Zealand jointly, and Ireland, and 1 study each was conducted in Belgium, Denmark, Malaysia, Spain and The Netherlands. These studies yielded 657 outcomes. Many of those outcomes were multiple presentations of same underlying concept (e.g. mortality had multiple presentations—at discharge, 30 days, 90 days, 1 year etc; disease-specific quality of life was represented in different ways depending on the underlying medical condition. Once these multiple representations were reduced to their underlying concepts, this yielded 76 distinct outcome concepts.

A total of 12 interviews and one focus group (including 8 members of the public) were conducted across the stakeholder groups. The interviews generated 64 outcomes, 7 of which were not covered by the concepts identified in the literature review. Details of the demographics of the interview and focus group participants are presented in Table 2.

**Table 2:** Interview (n = 12) and focus group (n = 8) sample demographics.

	Participants (n = 20)	
	Patient/public	= 8
	Surgeon/other clinical	= 6
	Service provider/evaluator	= 6
**Country**	UK	= 15
	Canada	= 2
	Europe	= 1
	Pakistan	= 1
	Africa	= 1

In summary, therefore, the literature review, interviews and focus group yielded, 83 outcomes which were taken forward for the Delphi survey. The outcomes were in four core areas: patient-level outcomes (37 outcomes), surgeon-level outcomes (17 outcomes), organisation-level outcomes (23 outcomes), and population-level outcomes (6 outcomes).

### International Delphi survey

169 participants (52 patient/public, 32 service provider/policy maker/researcher, and 85 surgeon/other clinical), and Round 2 was completed by 128 participants (43 patient/public, 27 service provider/policy maker/researcher, and 58 surgeon/other clinical). Details of the characteristics of the participants who completed both rounds are presented in Table 3.

**Table 3:** Delphi sample demographics.

	Participants (n = 128)		
**Sub-group**	Patient/public		= 43 (33.6%)
	Surgeon/other clinical		= 58 (45.3%)
	Service provider/evaluator		= 27 (21.1%)
**Gender**	Male = 72 (56.3%) Female = 55 (43.0%) Prefer not to say = 1 (0.8%)		
**Age (yrs)**	Under 18		= 0
	18–44		= 51 (39.8%)
	45–64		= 57 (44.5%)
	65–84		= 20 (15.6%)
	85 or more		= 0
**Country**	United Kingdom	= 89 (69.5%)	
	Italy	= 9 (7.0%)	
	Netherlands	= 7 (5.5%)	
	United States of America	= 4 (3.1%)	
	Greece	= 3 (2.3%)	
	India	= 3 (2.3%)	
	Turkey	= 3 (2.3%)	
	Australia	= 2 (1.6%)	
	Canada	= 2 (1.6%)	
	France	= 2 (1.6%)	
	Egypt	= 1 (0.8%)	
	Luxembourg	= 1 (0.8%)	
	Sweden	= 1 (0.8%)	
	Switzerland	= 1 (0.8%)	

Twenty-eight ‘new’ outcomes were considered for suggestion, and the study team took two of these forward for scoring in R2: surgeon satisfaction and environmental/carbon footprint. The other outcomes were rejected as being duplicates.

Following completion of Round 2, 12 outcomes achieved consensus for inclusion in principle in the COS at the whole group level, and 42 were excluded. Thirty-one outcomes did not reach consensus, and of these, 12 were taken forward for discussion at the consensus meeting. A further 4 outcomes that were classed as excluded at the whole group level, but which had wide variation in scoring between individual stakeholder groups, were also discussed at the consensus meeting. Details of all the R2 outcomes are reported in supplementary file S8 in S1 File Consensus Meeting Documents.

### Virtual/Online consensus meeting

The consensus meeting was attended by 14 participants (4 patient/ public, 4 surgeons/other clinical, and 6 service provider/evaluators) from the UK, Europe and Africa. Details of the characteristics of the consensus meeting participants are provided in Table 4.

**Table 4:** Consensus meeting demographics.

**Group**	Patient/public	= 4
	Surgeon/other clinical	= 4
	Service provider/evaluator	= 6
**Gender**	43% female	
**Country**	UK	= 12
	Europe	= 1
	Africa	= 1

The group was presented with the consensus ‘in’ outcomes and asked if there were any that should be discussed. The group decided that two outcomes ruled in from the Delphi should be discussed and re-voted. For the no consensus outcomes, the pre-determined rule for re-scoring was agreed (i.e. that 75% of all groups scoring 7–9 for patient level outcomes and 60% of all groups scoring 7–9 for all other outcomes) and 12 outcomes were taken forward for discussion. For the consensus ‘out’ outcomes 4 outcomes were taken forward for discussion and voting but were all re-voted as consensus out. Following voting on the no consensus and consensus out outcomes, the group reviewed the consensus ‘in’ outcomes for overlap, and three outcomes were discussed and re-scored. S10 Table in S1 File hows the group’s voting scores on the 21 outcomes discussed.

Of the 12 no consensus outcomes, 2 were voted “in” for the final COS (overall measure of complications, overall economic/cost-effectiveness). Reasons for ‘overall measure of complications’ being considered core included a belief that it was a more comprehensive measure than a procedure-specific outcome (and would include measures such as Clavien-Dindo scale which is comparable across settings, and surgical specialties) [17]. It was also noted that an overall measure for complications (such as Clavien-Dindo) would also include mortality and injuries and hence was a more holistic and comparable summary measure. It was also believed that a broader measure of complications, (such as Clavien-Dindo), would be easily relatable to economic data such as information on cost per complication to compare between surgeries.

Extending outcome measures for cost, the consensus meeting participants also voted ‘overall economic/cost-effectiveness’ in as a core outcome. There was discussion about its relevance for different geographical settings with representatives from LMICs reporting it as critically important. Given the capital cost of the intervention (the high procurement cost of a robotic system) it was believed being able to determine that the front-end investment was worthwhile, alongside patient and surgical outcomes, was critical.

Following voting of the agreed consensus out and no consensus outcomes, the group were asked to reconsider the Delphi-determined core outcome set to assess whether any existing outcomes overlapped with those now voted in. Based on this, four outcomes that had previously been agreed as consensus in from the Delphi were subsequently discussed and re-voted out of the final COS so as to avoid duplication. These were: Procedure-specific injury and Mortality (which were deemed as linked and they overlapped with the addition of ‘overall complications’ which would cover both). In addition, ‘surgeon autonomy’ was felt not to be specific enough and was also deemed to be covered by ‘standardisation of operative quality’ and therefore voted out. Finally, ‘Control of instruments’ was discussed and deemed to be interlinked and overlapping with precision/accuracy and as such voted out.

The final COS includes 10 outcomes (Table 5), consisting of four patient-level outcomes (disease-specific quality of life, overall quality of life, overall measure of treatment effectiveness/benefit, and overall measure of complications, including mortality), two surgeon-level outcomes (precision/accuracy, and visualisation), three organisation-level outcomes (equipment failure, standardisation of operative quality, and, overall economic/cost-effectiveness), and one population-level outcome (equity of access).

**Table 5:** Outcomes included in the final RoboCOS core outcome set.

Core Area	Outcome name	Description
Patient level	Overall-measure of complications inc. mortality	Overall measure of any adverse event resulting from the operation
	Overall measure of treatment effectiveness/benefit	How successful the procedure was overall
	Disease-specific quality of life	How well the patient feels physically and emotionally in relation to their specific health condition
	Overall quality of life	Overall state of the patient’s physical and mental wellbeing
Surgeon level	Precision/accuracy	The surgeon’s ability to carry out the procedure accurately without error
	Visualisation	The field of vision available to the surgeon during the procedure
Organisation level	Equipment failure	Any equipment failure
	Standardisation of operative quality	The degree to which variation in a given procedure is reduced and/or equal outcomes are achieved (e.g. reduced variation in implant alignment, or tumour access)
	Overall economic/cost-effectiveness	The value for money provided by a service, such as the cost-effectiveness of treatment route (medical management or surgery), calculated by dividing cost by success rate (defined by the quality of life after treatment)
Population level	Equity of access	Impact on the degree to which people have equal access to a given treatment or procedure

## Discussion

This the first study to develop a core outcome set for assessing the impact of robot assisted surgery across the whole system. It has highlighted a set of crucially important domains that should be addressed in any future evaluation of the technology and addresses a fundamental gap in many previous evaluations including its impact on the wider service and provision.

The identified core outcome set identifies a number of core outcomes at the clinical level including the importance of measuring complications and overall effect of treatment on quality of life. These have been widely used in previous trials to date showing the primary importance of measuring the impact of a new technology like robot-assisted surgery on patients [5]. Other outcomes have, however, been less widely used in evaluations to date. For example, little attention has been given in individual trials to date on the impact of RAS on either the surgeon, the organisation or the wider population. There have been certain notable exceptions–for example the UK NIHR-ROLARR study involved not only investigating the clinical impact of RAS [18], but also included a detailed assessment of the impact of the new technology on the surgeon and organisation [12]. Previous research has highlighted the impact of transformative technologies such as RAS on the surgeon and the organisation [19,20], and considered the impact on the wider population resulting from the decision to adopt, or not adopt, RAS, such as equity of access to healthcare. Routine assessment of its impact is thus crucial for future evaluations so that surgeons, service managers and policy makers can made fully informed decisions about its impact, both clinically, organisationally, and economically [21].

This study has several strengths. It had wide international input with international stakeholders contributing to the initial interviews, the Delphi survey and the final consensus meeting. Particularly relevant was the inclusion of views from low and middle income countries (LMICs) at all stages of the COS development, ensuring that the outcome are relevant across different healthcare settings. The COS development process also adopted international best-practice guidance and was pre-registered with the international COMET initiative. All outcome selection prior to the final consensus meeting was conducted blind to the outcome descriptors, ensuring no bias in the selection of what was to be ruled “in” or “out” for discussion.

The study does have some weaknesses. As, for convenience and efficiency, we used the existing systematic review by Garfjeld-Roberts *et al* [5] as the basis of our starter list of outcomes, rather than going back to the source documents, we are likely to have underestimated the number of distinct outcomes previous reported in trials (as the review has already effectively done some of the summarising of multiple outcome names for the same underlying concepts); however, it is unlikely that we have missed key concepts as this original review was augmented by additional literature searches and interviews. Additionally, although we had good international representation at all stages of the COS development, we have still only collated the views of a small range of individuals relative to the community who use and are considering the use of RAS. Lastly, we did not collect information on the level of expertise of the surgeons (or other stakeholders) included in the consensus process, the level of which may have influenced perspectives on importance of outcomes.

It is also important to note that whilst we have identified a small core set of outcomes which should be routinely used in evaluations, this does not mean that outcomes which are not in the core set are not important. Our Delphi survey demonstrated very high support for a wide range of outcomes, highlighting the importance of key elements, over and above those included in the final COS. As such, the final choice of the full spectrum of outcome to be measured in any specific future evaluation is likely to be much wider than the core set identified in this paper. Choice will be dependent of the research questions the evaluation wishes to answer and will likely involve additional highly-specific outcomes relevant to the particular clinical procedure being investigated and the relevant healthcare setting. It is also important to note that not all the outcomes can, or need, to be measured for each patient in a study. Measures such as standardisation of operative quality will, for example, be measured at the organisation level over a cohort of patients.

There are also a number of other core outcome sets developed for the assessment of surgical procedures which complement the RoboCOS core outcome set. For example, the COHESIVE core outcome set has been generated for the assessment of surgical procedures in the very early stages of innovation, may additionally apply for the development and evaluation of brand-new RAS systems and those in development [22].

Implementation of core outcome sets have been shown to improve the relevance of evaluations to all relevant stakeholders [23] and makes the future synthesis of evaluation findings easier, thus ensuring that the true impact of interventions can be assessed quicker and effective interventions identified earlier promoting their more rapid adoption. Implementation of the RoboCOS core outcome set should allow the outcomes of relevance to all the key stakeholders impacted by RAS systems to be identified and measured.

## Conclusion

The RoboCOS core outcome set, which includes outcomes of importance to all key stakeholders, is recommended for use in all future evaluations of robot-assisted surgery to ensure relevant and comparable reporting of outcomes.

## Supporting information

S1 File Search strategies. (DOCX)

S2 File Core Outcome Set-STandards for Reporting: The COS-STAR statement. (DOCX)
