# Supplementary material for: The RoboCOS Study: Development of an international core outcome set for the comprehensive evaluation of patient, surgeon, organisational and population level impacts of robotic assisted surgery
Source: PLoS One. 2023 Mar 30;18(3):e0283000. doi: 10.1371/journal.pone.0283000 (PMC10062593; doi:10.1371/journal.pone.0283000)
Supplement: S1 File — (DOCX) [file pone.0283000.s001.docx]

**S1 file – Search strategies**

Database: INHATA October 13^th^ 2020 (no date restriction)

Search Strategy: (robot) OR (robotics) OR (robotic) NOT (cyberknife) NOT (Cyberknife)

--------------------------------------------------------------------------------

1. robot (37)
2. robotics (3)
3. robotic (52)
4. 1 or 2 or 3 (92)
5. 4 NOT cyberknife (82)
6. 5 NOT Cyberknife (81)

**S2 file – Interview Methods and Topic Guide**

**Methods**

Participants were recruited from NHS staff. All participants gave verbal consent for their participation in a Microsoft Teams interview. Interviews were supported by a topic guide to ensure coverage of key issues and involved interviewees describing their opinion on service evaluation of robotic assisted surgery. All interviews were conducted by CR, audio-recorded, and transcribed verbatim.

**Interview Topic Guide:** **Robotic core outcome sets (RoboCOS): What outcomes are important for evaluating robotic assisted surgery as a service level change? An outcome mapping exercise to inform core outcome set development. Interview topic guide**

**Introduction (5-10 mins)**

Interviewer Name

Interviewer Occupation

Explain this study is interested in what outcomes people consider important for evaluating robotic assisted surgery (RAS) as a service level change. Explain scope: RAS in adults, for any health condition. Not considering other types of robotic assisted treatment such as radiotherapy/Cyberknife

The researcher will

• thank the interviewee for taking the time to attend

• explain the researcher is not a medical professional, want to understand the interviewee’s perspectives

• explain the duration of interview and confidentiality

• explain no right or wrong answers and participant can stop the interview at any time

• Researcher to confirm consent to qualitative interview with each potential participant

**Discussion and verbal consent**:

**With your permission, I would like to record our preliminary discussion about the study and the information in the consent form provided to you.**

- Check participant has had time to read consent form.
- Review essential aspects (i.e., purpose of study, what is being asked, confidentiality, how we will handle any concerns)
- Ask if there are any questions or concerns.
- Double check that all required boxes are checked and that participant has a signed copy of the consent form for their records.

**Start of Interview Questions**:

Confirm interviewee’s stakeholder group

Can you tell me about yourself?

Can you describe your particular areas of interest/expertise?

The interviews with stakeholders will of necessity be somewhat organic depending on the issues raised by the participants. However, each interview will seek to cover the following areas:

1. **Please tell me about your experience of RAS**

**What benefits do you think RAS confers over other services/interventions?**

**And what about the opposite of this – what do you think the risks are? or the potential challenges for use?**

**What do you think patients would be interested to know about RAS? Why?**

1. **If we were trying to determine if RAS was better or worse than existing procedures – how should we assess or evaluate that? What else?**
2. **Researcher introduces a list of outcomes generated from the literature review and explains a little bit about how the list of outcomes has been generated and the range of outcomes that have been identified.**

- When considering the types of results/outcomes that others have been perceived as being important when evaluating RAS
- As you are looking at the list of outcomes, can you pick a couple that you consider to be important and why?
- Can you tell me a little bit about how important you consider these outcomes in relation to one another? Which are more important than others?
- Are there any groups of people or situations where these outcomes might be less or more appropriate?
- In addition to this, how do you think these considerations would change over time?
- Are there any additional outcomes or indicators (whether measurable or not) that you think should be considered if we want to evaluate RAS?
  - Why? What is the reasoning about why these would matter/ be good?

1. **Any other issue the participant would like to raise**

**Thank you for your participation in our study.**

- Identify whether the participant wishes to be informed about the results of the overall project and if so are they happy with email contact.
- Check before ending the interview that participant gives permission to re-contact for clarification of issues arising in this interview

**Outcomes to explore in interviews as prompts**

Prompt the respondent to think about RAS at an individual patient, individual surgeon, team, organisational level, and any other groups of people or healthcare system levels throughout the interview

**S3 file – Focus Group Methods and Topic Guide**

**Methods**

Participants were recruited from an existing Patient and Public Involvement group.

All participants gave verbal consent for their participation in a Microsoft Teams interview. Interviews were supported by a topic guide to ensure coverage of key issues and involved interviewees describing their opinion on service evaluation of robotic assisted surgery. All interviews were conducted by CR, audio-recorded, and transcribed verbatim.

**Focus grou Topic Guide: Robotic core outcome sets (RoboCOS): What outcomes are important for evaluating robotic assisted surgery as a service level change? An outcome mapping exercise to inform core outcome set development. Focus group topic guide**

**Introduction (5-10 mins)**

Introductions from the focus group Chair, researcher and Chief Investigator - including names, occupations and role in the study

Chief Investigator will explain this study is interested in what outcomes people consider important for evaluating robotic assisted surgery (RAS) as a service level change. Will explain RAS definitions and meanings. Explain study scope: RAS in adults, for any health condition. Not considering other types of robotic assisted treatment such as radiotherapy/Cyberknife. The Chief Investigator will then leave the focus group.

The researcher will

- thank the focus group participants for taking the time to attend
- explain the researcher is not a medical professional, want to understand the focus group participants’ perspectives
- explain the duration of interview and confidentiality
- explain no right or wrong answers and participant can stop/leave focus group at any time
- Researcher to confirm consent to qualitative interview with each potential focus group participant

**Discussion and verbal consent** for each potential focus group participant

**With your permission, I would like to record our preliminary discussion about the study and the information in the consent form provided to you.**

- Check participant has had time to read consent form.
- Review essential aspects (i.e., purpose of study, what is being asked, confidentiality, how we will handle any concerns)
- Ask if there are any questions or concerns.
- Double check that all required boxes are checked and that participant has a signed copy of the consent form for their records.

**Start of Interview Questions**:

Confirm focus group participant’s stakeholder group – can you tell me about yourself

The interviews with stakeholders will of necessity be somewhat organic depending on the issues raised by the participants. However, each interview will seek to cover the following areas:

1. **Can you tell me what you think of when you think about robotic assisted surgery and/or Have you or anyone you know ever had one of the RAS procedures?**
2. **How would you feel if you were told you were going to have a robotic assisted procedure?**
   1. **Why would you feel that way?**
3. **What would you want to know about?**
4. **Would you decline to have robotic assisted surgery?**
   1. **Why/Why not?**
5. **What benefits do you think RAS confers over other services/interventions?**
6. **And what about the opposite of this – what do you think the risks are? or the potential challenges for use?**

**What do you think patients would be interested to know about RAS? Why?**

1. **If we were trying to determine if RAS was better or worse than existing procedures – how should we assess or evaluate that? What else?**
2. **Researcher introduces a list of outcomes generated from the literature review and explains a little bit about how the list of outcomes has been generated and the range of outcomes that have been identified.**

- When considering the types of results/outcomes that others have been perceived as being important when evaluating RAS
- As you are looking at the list of outcomes, can you pick a couple that you consider to be important and why?
- Can you tell me a little bit about how important you consider these outcomes in relation to one another? Which are more important than others?
- Are there any groups of people or situations where these outcomes might be less or more appropriate?
- In addition to this, how do you think these considerations would change over time?
- Are there any additional outcomes or indicators (whether measurable or not) that you think should be considered if we want to evaluate RAS?
  - Why? What is the reasoning about why these would matter/ be good?

1. **Any other issue the participant would like to raise**

**Thank you for your participation in our study.**

- Identify whether the participant wishes to be informed about the results of the overall project and if so are they happy with email contact.
- Check before ending the interview that participant gives permission to re-contact for clarification of issues arising in this interview

**Outcomes to explore in interviews as prompts**

Prompt the respondent to think about RAS at an individual patient, individual surgeon, team, organisational level, and any other groups of people or healthcare system levels throughout the interview

**S4 File – Outcome Reduction**

| **Level** | **Delphi domain** | **Delphi outcome** | **Verbatim outcome from literature** | **Verbatim outcome from interviews** | **Verbatim outcome from public (focus group)** |
| --- | --- | --- | --- | --- | --- |
| **Patient (n=433)** | Intraoperative complications (n=26) | Procedure-specific injury (n=21) | New-onset incarcerated inguinal hernia |  |  |
|  |  |  | Injury | Injury |  |
|  |  |  |  | Insult to patient |  |
|  |  |  | Organ injury (perioperative adverse event) |  |  |
|  |  |  |  | Soft tissue damage |  |
|  |  |  | Enterotomy |  |  |
|  |  |  | Caecal damage |  |  |
|  |  |  | Internal iliac artery injury |  |  |
|  |  |  | Nerve injury |  |  |
|  |  |  | Neurologic event |  |  |
|  |  |  | vessel injury |  |  |
|  |  |  | Femoral nerve injury |  |  |
|  |  |  | Damage to peripheral organs |  |  |
|  |  |  | rectal & small bowel injury |  |  |
|  |  |  | Ureteral injury |  |  |
|  |  |  | Femoral nerve injury |  |  |
|  |  |  | Obturator nerve injury |  |  |
|  |  |  | rectal damage/injury |  |  |
|  |  |  | Ureteric injury |  |  |
|  |  |  | Injury of vena cava |  |  |
|  |  |  | Brachial plexus injury |  |  |
|  |  | Blood loss/transfusion (n=5) | Blood loss | Blood loss | Blood loss |
|  |  |  | Estimated blood loss |  |  |
|  |  |  | Blood transfusion | Blood transfusion |  |
|  |  |  | Transfusion intra-operative |  |  |
|  |  |  | Transfusion post-operative |  |  |
|  | Post-operative complications (n=169) | General measure (n-17) | Adverse events |  |  |
|  |  |  |  | Adverse clinical outcomes |  |
|  |  |  | Clavien-Dindo score | Clavien-Dindo score |  |
|  |  |  | Morbidity |  |  |
|  |  |  | Minor complication rate |  |  |
|  |  |  | Major complication rate |  |  |
|  |  |  | Complications | Complications |  |
|  |  |  |  | Near miss events |  |
|  |  |  | Perioperative hematocrit |  |  |
|  |  |  | Change in hematocrit |  |  |
|  |  |  | Acute phase reaction |  |  |
|  |  |  | Post-operative hemorrhage |  |  |
|  |  |  | adhesion formation |  |  |
|  |  |  | anemia |  |  |
|  |  |  | Superficial phlebitis |  |  |
|  |  |  | Decubitus ulcer |  |  |
|  |  |  | pelvic adhesions |  |  |
|  |  | Infection (n=19) | Infection | Infection |  |
|  |  |  |  | Infection prevention - COVID-19 |  |
|  |  |  | Infection rate | Infection rate | Infection |
|  |  |  | Wound infection | Wound infection |  |
|  |  |  | Wound infection rate |  |  |
|  |  |  | Infected hematoma |  |  |
|  |  |  | Peritonitis |  |  |
|  |  |  | acute peritonitis |  |  |
|  |  |  | abdominal abscess |  |  |
|  |  |  | Parietal abscess |  |  |
|  |  |  | Fever |  |  |
|  |  |  | Arm cellulitis |  |  |
|  |  |  | cellulitis |  |  |
|  |  |  | Sepsis |  |  |
|  |  |  | c. difficile colitis |  |  |
|  |  |  | Thrush |  |  |
|  |  |  | Urinary tract infection |  |  |
|  |  |  | (infected) lymphocele |  |  |
|  |  |  | Lymphedema |  |  |
|  |  | Wound-related (n=6) | Rates of wound complications |  |  |
|  |  |  | Wound dehiscence |  |  |
|  |  |  | seroma |  |  |
|  |  |  | Port hernia |  |  |
|  |  |  | Incisional hernia |  |  |
|  |  |  | Umbilical hernia |  |  |
|  |  | procedure-specific pain (5) | Pain | Pain | Pain |
|  |  |  | joint pain |  |  |
|  |  |  | Postoperative pain at 14 days |  |  |
|  |  |  | Postoperative pain at 3 days |  |  |
|  |  |  | Pain at 2 weeks |  |  |
|  |  | Cognitive (n=3) | Confusion |  |  |
|  |  |  | Altered mental state |  |  |
|  |  |  | Insomnia/sleep disturbance |  |  |
|  |  | Cerebrovascular (n=5) | Transient ischaemic attack |  |  |
|  |  |  | Stroke at 30 days |  |  |
|  |  |  | Stroke at 1 year |  |  |
|  |  |  | stroke |  |  |
|  |  |  | compartment syndrome |  |  |
|  |  | Cardiovascular (n=16) | Cardiovascular complication |  |  |
|  |  |  | Myocardial infarction |  |  |
|  |  |  | Myocardial infarction at 30 days |  |  |
|  |  |  | other cardiovascular complications |  |  |
|  |  |  | pacemaker implantation |  |  |
|  |  |  | Arrhythmia |  |  |
|  |  |  | post-operative atrial fibrillation |  |  |
|  |  |  | Thromboembolism |  |  |
|  |  |  | Pseudoaneurysm |  |  |
|  |  |  | Venous gas embolism |  |  |
|  |  |  | hypertension |  |  |
|  |  |  | atrial fibrillation |  |  |
|  |  |  | Peripheral embolus |  |  |
|  |  |  | Deep vein thrombosis (perioperative adverse event) |  |  |
|  |  |  | Superficial phlebitis |  |  |
|  |  |  | residual mitral regurgitation severity |  |  |
|  |  | Renal (n=5) | Acute renal failure |  |  |
|  |  |  | Post-surgical renal failure |  |  |
|  |  |  | renal failure |  |  |
|  |  |  | Dehydration |  |  |
|  |  |  | Glomerular filtration rate |  |  |
|  |  | Pulmonary/respiratory (n=5) | Pneumonia |  |  |
|  |  |  | Pulmonary pleural effusion |  |  |
|  |  |  | pulmonary insufficiency |  |  |
|  |  |  | Pulmonary embolism (perioperative adverse event) |  |  |
|  |  |  | pulmonary event |  |  |
|  |  | Urinary (n=28) | Retention/clot retention |  |  |
|  |  |  | Urinary retention |  |  |
|  |  |  | Post-catheter retention |  |  |
|  |  |  | Urinoma |  |  |
|  |  |  | Delayed voiding |  |  |
|  |  |  | Bladder lithiasis |  |  |
|  |  |  | Bladder neck contracture/stenosis (perioperative adverse event) |  |  |
|  |  |  | Upper urinary tract complication |  |  |
|  |  |  | Urinary fistula |  |  |
|  |  |  | Urinary leak |  |  |
|  |  |  | Ureteral transection (intraoperative |  |  |
|  |  |  | Post-operative incontinence |  |  |
|  |  |  | Urinary dysfunction |  |  |
|  |  |  | Urinary incontinence |  |  |
|  |  |  | 1-year urinary incontinence |  |  |
|  |  |  | Urinary (In(continence rate (leak/no leak) 3 months |  |  |
|  |  |  | Urinary (In(continence rate (leak/no leak) 12 months |  |  |
|  |  |  | Urinary function 3 months |  |  |
|  |  |  | Urinary function 6 months |  |  |
|  |  |  | Urinary function 12 months |  |  |
|  |  |  | Urinary continence |  |  |
|  |  |  | Urinary continence at 3 months |  |  |
|  |  |  | Urinary continence at 6 months |  |  |
|  |  |  | Urinary continence at 12 months |  |  |
|  |  |  | Recovery of urinary continence at 6 months |  |  |
|  |  |  | Contienence at 6 months |  |  |
|  |  |  | Contienence at 12 months |  |  |
|  |  |  | Continence | Continence |  |
|  |  | Uterovaginal (n=10) | Vaginal cut dehiscence |  |  |
|  |  |  | Vaginal access |  |  |
|  |  |  | vaginal cuff hematoma |  |  |
|  |  |  | vaginal seroma |  |  |
|  |  |  | vaginal leak |  |  |
|  |  |  | rectovaginal fluid collection/abscess |  |  |
|  |  |  | Ureterovaginal fistula |  |  |
|  |  |  | vaginal bleeding |  |  |
|  |  |  | uterine rupture |  |  |
|  |  |  | re-anastomosis |  |  |
|  |  | Gastrointestinal (n=20) | Gastrointestinal stoma creation |  |  |
|  |  |  | Biliary colic/cholecystitis |  |  |
|  |  |  | Enterocutaneous fistula |  |  |
|  |  |  | ileus |  |  |
|  |  |  | Delayed ileus |  |  |
|  |  |  | Prolonged ileus |  |  |
|  |  |  | Colonic pseudo-obstruction |  |  |
|  |  |  | stomal stenosis |  |  |
|  |  |  | Conduit obstruction |  |  |
|  |  |  | partial small bowel obstruction |  |  |
|  |  |  | endoscopic eosophagitis (fundoplication) |  |  |
|  |  |  | Anastomic leak |  |  |
|  |  |  | Anastomosis leakage |  |  |
|  |  |  | Anastomtic stenosis |  |  |
|  |  |  | Anastomotic stricture |  |  |
|  |  |  | Number of anastomotic strictures |  |  |
|  |  |  | Anastomosis time |  |  |
|  |  |  | dysphagia at 1 year (fundoplication) |  |  |
|  |  |  | dysphagia at 30 days (fundoplication) |  |  |
|  |  |  | dysphagia post op (fundoplication) |  |  |
|  |  | Head and neck (n=8) | neck hematomas |  |  |
|  |  |  | pharyngocutaneous fistula |  |  |
|  |  |  | Dysphagia |  |  |
|  |  |  | post-operative regurgitation |  |  |
|  |  |  | transient hoarseness |  |  |
|  |  |  | RLN palsy |  |  |
|  |  |  | moderate trismus |  |  |
|  |  |  | mucosal bleeding |  |  |
|  |  | Urology (n=1) | Epididymitis |  |  |
|  |  | Endocrine (n=2) | transient hypocalcaemia |  |  |
|  |  |  | calcium and parathyroid hormone levels |  |  |
|  |  | Functional (n=19) | Post-prostatectomy potency |  |  |
|  |  |  | Post-operative sexual function |  |  |
|  |  |  | Impotence |  |  |
|  |  |  | Erectile function/dysfunction | Erectile function/dysfunction |  |
|  |  |  | Capability for intercourse |  |  |
|  |  |  | erectile function 3 months |  |  |
|  |  |  | erectile function 6 months |  |  |
|  |  |  | erectile function 12 months |  |  |
|  |  |  | Erection sufficient for intercourse at 3 months |  |  |
|  |  |  | Sexual function after prostatectomy/ability to maintain an erection after 12 months |  |  |
|  |  |  | Return to erection at 12 months |  |  |
|  |  |  | 1-year erectile function |  |  |
|  |  |  | Recovery of erectile function |  |  |
|  |  |  | Recovery of sexual potency |  |  |
|  |  |  | Return of sexual potency |  |  |
|  |  |  | Sexual function |  |  |
|  |  |  | Female sexual function at 6 months |  |  |
|  |  |  | Sexual function at 3 months |  |  |
|  |  |  | Sexual function at 12 months |  |  |
|  | Length of ICU stay (n=1) |  | length of ICU stay |  |  |
|  | Length of hospital stay (n=9) |  | Length of (hospital) stay | Length of (hospital) stay |  |
|  |  |  |  |  | People stay in hospital for less time |
|  |  |  | Postoperative hospital stay |  |  |
|  |  |  | Mean total length of stay |  |  |
|  |  |  | Annual reduction in bed days |  |  |
|  |  |  | Gain in bed days |  |  |
|  |  |  | Time to discharge |  |  |
|  |  |  | Proportion discharged within 24 hours |  |  |
|  |  |  | Cost of length of stay | Cost of length of stay |  |
|  | Treatment effectiveness (n=39) | Operative progress (n=17) | in-house analgesia usage |  |  |
|  |  |  |  | they didn't require as much analgesia |  |
|  |  |  | Time to removal of catheter/duration of catheterisation |  |  |
|  |  |  | Drain duration |  |  |
|  |  |  | Intubation time |  |  |
|  |  |  | Ventilation time |  |  |
|  |  |  | Conversion rates |  |  |
|  |  |  | Conversion to open rate |  |  |
|  |  |  | Number of conversions |  |  |
|  |  |  | Conversion to open surgery | Conversion to open surgery |  |
|  |  |  | Conversion to open rates |  |  |
|  |  |  | Conversion to manual technique |  |  |
|  |  |  | Conversion to conventional operation |  |  |
|  |  |  | Conversion to radical nephrectomy |  |  |
|  |  |  | Conversion to laparotomy |  |  |
|  |  |  | Conversion to laparoscopic or open | Conversion to laparoscopic or open |  |
|  |  |  | Conversion cost |  |  |
|  |  | General measure (n=1) |  | Long-term impacts on patient | Long(er)-term impacts on patient |
|  |  | Overall measure of treatment effectiveness/benefit (n=21) | Stone disintegration, Stone free at 3 months, residual fragments |  |  |
|  |  |  | oesophageal sphincter resting pressure (fundoplication) |  |  |
|  |  |  | post-operative pH values (fundoplication) |  |  |
|  |  |  | contrast swallow control (fundoplication) |  |  |
|  |  |  | mean reflux score |  |  |
|  |  |  | aspiration or velopharyngeal reflux |  |  |
|  |  |  | Reflux symptoms at 6 months |  |  |
|  |  |  | Weight loss |  |  |
|  |  |  | Insulin resistance |  |  |
|  |  |  | Fertility pregnancy/ectopic pregnancy/spontaneous abortion rates |  |  |
|  |  |  | Sternotomy (avoidance of) |  |  |
|  |  |  | Aseptic loosening |  |  |
|  |  |  | Heterotopic ossification |  |  |
|  |  |  | Renal pelvis AP diameter change |  |  |
|  |  |  | long-term durability of repair |  |  |
|  |  |  | Accuracy of screw placement |  |  |
|  |  |  | Anatomic outcome |  |  |
|  |  |  | anatomical result of fundoplication |  |  |
|  |  |  | Implant position |  |  |
|  |  |  | Radiological accuracy |  |  |
|  |  |  | Gait kinematics |  |  |
|  | Quality of life generic (n=10) |  | Quality of life (generic) | Quality of life (generic) |  |
|  |  |  | Return to normal physical component of HRQOL scores |  |  |
|  |  |  | QOL at 1 month |  |  |
|  |  |  | QOL 3 months post surgery |  |  |
|  |  |  | QOL at 2 weeks, 6 weeks and 3 months |  |  |
|  |  |  | HRQOL at 12 months |  |  |
|  |  |  | SF-36 |  |  |
|  |  |  | EQ-5D |  |  |
|  |  |  | SF-12 |  |  |
|  |  |  | HADS |  |  |
|  | Quality of life disease-specific (n=42) |  | Quality of life (disease-specific) | Quality of life (disease-specific) |  |
|  |  |  | Treatment goals: regaining urinary control following prostatectomy |  |  |
|  |  |  | Incontinence related quality of life at 12 months |  |  |
|  |  |  | Prostate symptom score/IPS |  |  |
|  |  |  | Prostate cancer distress |  |  |
|  |  |  | Self-rated change in symptoms/oesophagitis from pre-operative state at 1 month |  |  |
|  |  |  | Self-rated change in symptoms/oesophagitis from pre-operative state at 3 month |  |  |
|  |  |  | Self-rated change in symptoms/oesophagitis from pre-operative state at 12 month |  |  |
|  |  |  | Symptom relief |  |  |
|  |  |  | Improvement in GERD activity index (GRACI) |  |  |
|  |  |  | mean reflux score |  |  |
|  |  |  | Functional hip score at 3 months |  |  |
|  |  |  | Functional hip score at 6months |  |  |
|  |  |  | Functional hips score at 12 months |  |  |
|  |  |  | Functional hip score at 24 months |  |  |
|  |  |  | Pelvic tilt |  |  |
|  |  |  | Pelvic obliquity |  |  |
|  |  |  | Hip flexion/extension |  |  |
|  |  |  | Hip abduction/adduction |  |  |
|  |  |  | Ossification (enhanced) |  |  |
|  |  |  | Voice quality and articulation at 1 month |  |  |
|  |  |  | OKS |  |  |
|  |  |  | AKSS American Knee Society Score |  |  |
|  |  |  | Forgotten Joint Score |  |  |
|  |  |  | Pain Catastrophising Score |  |  |
|  |  |  | Pain VAS, pain scores, Analgeisc use etc |  |  |
|  |  |  | Stiffness VAS |  |  |
|  |  |  | Range of motion |  |  |
|  |  |  | UCLA Activity Scale |  |  |
|  |  |  | EPIC |  |  |
|  |  |  | IIEF |  |  |
|  |  |  | REIS |  |  |
|  |  |  | ODI (Oswestry Disability Index) |  |  |
|  |  |  | Oswestry Disability Index at 1 year |  |  |
|  |  |  | FACT-VCI |  |  |
|  |  |  | WOMAC |  |  |
|  |  |  | Japanese Orthopaedic Association Score |  |  |
|  |  |  | MDADI (swallowing related quality of life) |  |  |
|  |  |  | EPH-30 |  |  |
|  |  |  | POP-Q |  |  |
|  |  |  | Pelvic Floor Distress Inventory |  |  |
|  |  |  | International prostate symptom score (IPSS) reduction |  |  |
|  | Time to recovery (n=22) | Physical (n=15) | Recovery time | Recovery time | Recovery time |
|  |  |  | Time to flatus |  |  |
|  |  |  | Time to bowel movement |  |  |
|  |  |  | Return to function (swallowing) |  |  |
|  |  |  | Elimination of sleep apnea |  |  |
|  |  |  | Time to return to continence |  |  |
|  |  |  | Time to urinary continence 3 months |  |  |
|  |  |  | Time to urinary continence 6 months |  |  |
|  |  |  | Time to urinary contience 12 months |  |  |
|  |  |  | Time to recovery of erectile function/potency |  |  |
|  |  |  | Time to recovery of sexual function |  |  |
|  |  |  | Time to recovery of sexual intercourse |  |  |
|  |  |  | Days to oral intake |  |  |
|  |  |  | Days to regular diet |  |  |
|  |  |  | alimentation days (fundoplication) |  |  |
|  |  | Role (n=7) | Productivity - lost productivity up to 52 days post discharge |  |  |
|  |  |  | Productivity - time to return to full activity or work |  |  |
|  |  |  | Time to return to daily activities or work |  |  |
|  |  |  | Time to mobilsation or return to work or activity |  |  |
|  |  |  | Return to daily activities or work (rate) |  |  |
|  |  |  | Time away from employment |  |  |
|  |  |  | Return to work |  |  |
|  | Patiet perception of RAS (n=10) |  | Patients require education, information and support - A key theme running throughout the studies was that patients require more information and support when it comes to RS and decision-making around surgery options |  |  |
|  |  |  | Who provides information and support for decision-making - Among men diagnosed with prostate cancer, there was considerable uncertainty and decision-related distress around treatment options, and participants explained that pre-operative education, information, and support from surgeons, nurses, as well as other patients who have experienced RS is a necessity |  |  |
|  |  |  | Minimally invasive - The minimally invasive nature of RARP compared to open RP influenced the themes surrounding the entire experience | Minimally invasive |  |
|  |  |  | Female versus male opinions - safety, trust and de-humanising/anthropomorphism - The majority of female participants expressed concerns in relation with the safety and perception of RS, whereas many male participants appeared to be untroubled by the idea of RS. The lack of acceptance expressed by most female participants appeared to be based on trust. |  |  |
|  |  |  | Safety/trust concerns with new technology |  |  |
|  |  |  |  |  | Is the technology safe |
|  |  |  | De-humanising v anthropomorphism - While female participants viewed RS as de-humanizing, males humanized surgical robots and exhibited a sense of anthropomorphism in relation with RS |  |  |
|  |  |  | Misconceptions/how robot works/Lack of understanding around surgeon's role/who is in control of the operation | Misconceptions about how the robot works |  |
|  |  |  | Media reporting (information v misconception) - the media were described as an important source of information for both male and female participants in relation to attitudes towards the acceptance of RS. For some participants, the media were an effective tool in increasing awareness and understanding and acceptance, but for others, the media resulted in misconceptions about RS |  |  |
|  |  |  |  | Perception of robots in films | Perception of robots in movies and books |
|  | Patient experience (21) | Emotion (6) | Psychological distress |  |  |
|  |  |  | Mental health | Mental health |  |
|  |  |  | Self-esteem |  |  |
|  |  |  |  | Patient apprehension - of new technology |  |
|  |  |  |  | Patient enthusiasm - of new technology |  |
|  |  |  | Psychosocial impact/personal identity - Following RALP, men considered themselves ‘lucky’ to be alive and re-evaluated their lives particularly with regards to their relationships and future goals |  |  |
|  |  | Patient-surgeon-nurse relationship (5) | relationships |  |  |
|  |  |  |  | Communication with surgeon/team | Communication with surgeon |
|  |  |  |  | Patient trust in surgeon |  |
|  |  |  | Quality of informed consent | Quality of informed consent |  |
|  |  |  |  | The patient-surgeon relationship |  |
|  |  | Satisfaction general (n=4) | Patient satisfaction | Patient satisfaction |  |
|  |  |  | Overall satisfaction |  |  |
|  |  |  | Likely to recommend to others |  |  |
|  |  |  |  | Satisfaction related to the status associated with having a type of procedure |  |
|  |  | Cosmesis (4) |  | Size of scar (smaller scar) | Size of scar (smaller scar) |
|  |  |  | Satisfaction with body image |  |  |
|  |  |  | Satisfaction with surgical scar |  |  |
|  |  |  | Cosmetic advantage |  |  |
|  |  | Satisfaction with procedure-specific outcome (2) | Self-reported satisfaction with change in condition |  |  |
|  |  |  | Self-reported satisfaction with operative result |  |  |
|  | Readmission (n=18) | Post-operative readmission/reoperation (n=18) | abdominal pain requiring readmission |  |  |
|  |  |  | Reoperation rate |  |  |
|  |  |  | Reoperations | Reoperations |  |
|  |  |  | Revision rate |  |  |
|  |  |  | Readmission rate |  |  |
|  |  |  | Surgical reintervention |  |  |
|  |  |  |  |  | Further intervention |
|  |  |  | Reoperation rate within 30 days |  |  |
|  |  |  | Reoperation due to bleeding at 30 days |  |  |
|  |  |  | Reoperation for bleeding |  |  |
|  |  |  | Bleeding requiring reoperation |  |  |
|  |  |  | Secondary surgery for incontinence |  |  |
|  |  |  | Tracheotomy |  |  |
|  |  |  | Tracheostomy |  |  |
|  |  |  | Readmissions/rate |  |  |
|  |  |  | Unscheduled clinic visits |  |  |
|  |  |  | Cost of readmission |  |  |
|  |  |  |  | Cost of reoperation/revision |  |
|  | Disease progression/treatment failure (n=17) |  | Biochemical (PSA) recurrence/failure |  |  |
|  |  |  | Biochemical (PSA) recurrence-free rate |  |  |
|  |  |  | Biochemical free survival rate/actuarial biochemical free survival rate |  |  |
|  |  |  | Biochemical failure rate |  |  |
|  |  |  | Rising PSA |  |  |
|  |  |  | PSA relapse rate |  |  |
|  |  |  | PSA recurrence |  |  |
|  |  |  | PSA detection during follow-up |  |  |
|  |  |  | PSA level |  |  |
|  |  |  | Postoperative PSA |  |  |
|  |  |  | Detectable PSA rate |  |  |
|  |  |  | Further cancer treatment |  |  |
|  |  |  | Time to adjuvant chemotherapy |  |  |
|  |  |  | Cancer recurrence |  |  |
|  |  |  | Recurrence rate |  |  |
|  |  |  | Prolapse recurrence |  |  |
|  |  |  | 3-year local disease recurrence |  |  |
|  | Subsequent treatment (n=2) |  | Adjuvant or salvage radiation |  |  |
|  |  |  | Reduced morbidity from lower chemoradiation |  |  |
|  | Treatment affordability (n=1) |  |  | Can the patient afford the treatment/How financially affordable the operation is for the patient |  |
|  | Mortality (n=46) | Mortality (n=9) | Mortality |  |  |
|  |  |  | Morality rate |  |  |
|  |  |  | All-cause mortality | All-cause mortality |  |
|  |  |  | In-hospital mortality |  |  |
|  |  |  | 30-day mortality |  |  |
|  |  |  | 60-day mortality |  |  |
|  |  |  | 90-day mortality |  |  |
|  |  |  | Perioperative mortality |  |  |
|  |  |  | Postoperative mortality |  |  |
|  |  | Death (n=5) | Deaths |  |  |
|  |  |  | Death rate |  |  |
|  |  |  | Cardiac related death at 1 year |  |  |
|  |  |  | Intraoperative death |  |  |
|  |  |  | Death secondary to aspiration |  |  |
|  |  | Survival/disease-free survival (n=9) | Disease-free survival |  |  |
|  |  |  | Disease-specific survival rate |  |  |
|  |  |  | Survival |  |  |
|  |  |  | Survival rate |  |  |
|  |  |  | 30-day survival |  |  |
|  |  |  | 5-year survival |  |  |
|  |  |  | Recurrence free survival |  |  |
|  |  |  | 3-year overall survival |  |  |
|  |  |  | Cancer-specific survival |  |  |
|  |  | Proxy measure - cancer margins (n=12) | surgical resection |  |  |
|  |  |  | Positive surgical margins |  |  |
|  |  |  | Margin status |  |  |
|  |  |  | Tumour free margin |  |  |
|  |  |  | Proximal margin |  |  |
|  |  |  | Distal margin |  |  |
|  |  |  | circumferential resection margin positivity |  |  |
|  |  |  | proximal resection margins |  |  |
|  |  |  | Distal resection margins |  |  |
|  |  |  | Circumferential radial margin |  |  |
|  |  |  | Benign prostate glands at bladder neck margin |  |  |
|  |  |  | Quality of TME (pathologist graded, as per Nagtegaal) |  |  |
|  |  | Proxy measure - cancer other (n=11) | Pathologic stage |  |  |
|  |  |  | Lymph nodes |  |  |
|  |  |  | Lymph node yield |  |  |
|  |  |  | Detected lymph nodes |  |  |
|  |  |  | Lymph node retrieval |  |  |
|  |  |  | Number of lymph nodes recovered |  |  |
|  |  |  | Mean number of lymph nodes harvested |  |  |
|  |  |  | Harvested lymph nodes |  |  |
|  |  |  | Lymphovascular invasion |  |  |
|  |  |  | Gleason score |  |  |
|  |  |  | distant metastasis |  |  |
| **Surgeon (n=97)** | Learning curve (n=2) | Learning curve (n=1) | Learning curve | Learning curve |  |
|  |  | Maintaining skill/competency (adequate access to robot) (n=1) |  | Maintaining skill/competency (adequate access to robot) |  |
|  | Surgeon motivation (n=7) | Surgeon confidence in team (n=1) | Surgeon confidence in team |  |  |
|  |  |  |  | Risk-taking attitude |  |
|  |  |  | Surgeon sense of pride or excitement at the new innovation in their organization |  |  |
|  |  |  | Surgeon/Team enthusiasm at the opportunity to learn new technologies | Surgeon/Team enthusiasm at the opportunity to learn new technologies |  |
|  |  |  | Surgeon hopefulness for the potential enhanced functions of RAS compared to laparoscopic and open surgery |  |  |
|  |  |  | Motivation of users |  |  |
|  |  |  | Surgeon willingness to undertake operation | Surgeon willingness to undertake operation |  |
|  | Operative control (n=18) |  | Haptic and tactile feedback |  |  |
|  |  |  | Haptics |  |  |
|  |  |  | Haptic feedback |  |  |
|  |  |  | Tactile feedback |  |  |
|  |  |  | Tactile sensation |  |  |
|  |  |  | Lack of tactile feedback/reliance on visual cues |  |  |
|  |  |  | articulation of instruments | articulation of instruments |  |
|  |  |  | Durability of instruments |  |  |
|  |  |  | Increased control |  |  |
|  |  |  | (Better) operative control |  |  |
|  |  |  | Control of retraction (awareness of instruments) |  |  |
|  |  |  | (Better) dexterity | (Better) dexterity |  |
|  |  |  | Rotation and range of motion of device arms | Rotation and range of motion of device arms |  |
|  |  |  | (Elimination of) hand tremour |  |  |
|  |  |  | Better visualisation | (Better) visualisation |  |
|  |  |  | Precision/accuracy | Precision/accuracy |  |
|  |  |  | Surgeon autonomy | Surgeon autonomy |  |
|  |  |  |  |  | degree of [surgeon] control |
|  | Operative time (n=17) |  | Operation time | Operation time | Operation time |
|  |  |  | Longer operative durations due to robot set-up, docking, patient positioning |  |  |
|  |  |  | Time patient in operating room (cost of) |  |  |
|  |  |  | Cost of recovery room time |  |  |
|  |  |  | Operative time skin-to-skin |  |  |
|  |  |  | Operative time - time in room |  |  |
|  |  |  | Console time |  |  |
|  |  |  | Console time plus set up |  |  |
|  |  |  | Patient set up time | Patient set up time |  |
|  |  |  | Docking time |  |  |
|  |  |  | time from insertion of Foley catheter to closing of last trocar site |  |  |
|  |  |  | time from the start of first side wall to vaginal cuff closure |  |  |
|  |  |  | bypass time |  |  |
|  |  |  | bypass plus aortic cross-clamp time |  |  |
|  |  |  | time to extubation |  |  |
|  |  |  | Time to prepare (assistant) |  |  |
|  |  |  | Surgeon speed |  |  |
|  | Physical impact/surgeon ergonomics (n=13) | Comfort/physical strain (n=10) | Surgeon morbidity | Surgeon morbidity |  |
|  |  |  | Neck strain - surgeon |  |  |
|  |  |  | Shoulder strain - surgeon |  |  |
|  |  |  | Hand strain - surgeon |  |  |
|  |  |  | Upper back strain - surgeon |  |  |
|  |  |  |  | Repetitive strain injuries |  |
|  |  |  | Ergonomic positioning of arm |  |  |
|  |  |  | Ergonomic positioning of wrist |  |  |
|  |  |  | Ergonomic positioning of trunk |  |  |
|  |  |  | Surgeon discomfort/comfort | Surgeon discomfort/comfort |  |
|  |  | Fatigue (n=1) | Surgeon fatigue | Surgeon fatigue |  |
|  |  | Nausea/vertigo (1) |  | Surgeon nausea/vertigo |  |
|  |  | length of surgeon's working life (1) |  | Operating lifespan of the surgeon |  |
|  | Emotional/cognitive impact (n=10) | Emotional impact (n=4) | Stress of performing RAS |  |  |
|  |  |  | Surgeon stress/workload | Surgeon stress/workload | Surgeon stress/workload |
|  |  |  |  | Dysjunction from patient (emotional disconnect -easier to operate) |  |
|  |  |  |  | Emotional fatigue |  |
|  |  | Cognitive impact (n=1) | Decision making quality (of surgeon) |  |  |
|  |  | Impact on concentration (n=5) | Surgeon focus |  |  |
|  |  |  | Distaction (of surgeon) |  |  |
|  |  |  | Concentration (of surgeon) |  |  |
|  |  |  | Heightened concentration compared with lap/open |  |  |
|  |  |  | Co-ordination - surgeon is separated from the rest of the OT team |  |  |
|  | Situational awareness (n=2) |  | Sense of immersion causing reduced situational awareness - A strategy to address this described by operating room (OR) teams in one study was positioning the console so that the surgeon has a direct view of the patient and the assistant when they look up from the robot |  |  |
|  |  |  | Situational awareness / surgeon's position at the console |  |  |
|  | Quality of communication in the operative team (27) |  | Partnering surgeons- Some surgeon participants stated that they shared the operation with a colleague and this strategy reduced their levels of stress around performing RS |  |  |
|  |  |  | Team communication and trust -Good team communication and team trust were seen as essential parts of robotic surgery. Because the surgeon is seated behind the console separate from the rest of the surgical team, team trust and communication between the team and the surgeon is more important in RS than in laparoscopic or open surgery. The surgeon has to rely on the rest of the team to communicate information outside of their field of vision to avoid complications, reduce distraction, and increase concentration. Surgeons require communication about both the state of the patient and the state of the robot. |  |  |
|  |  |  | Positive relationship between surgeon and team |  |  |
|  |  |  | Dedicated teams based on interest/enthusiasm, experience/skill | Dedicated teams based on interest/enthusiasm, experience/skill |  |
|  |  |  | Communication |  |  |
|  |  |  | Decision-making in the operating theatre |  |  |
|  |  |  | Team experience |  |  |
|  |  |  | Team involvement |  |  |
|  |  |  | Team motivation |  |  |
|  |  |  | Team communication | Team communication |  |
|  |  |  | Team understanding/support | Team understanding/support |  |
|  |  |  | Team coordination |  |  |
|  |  |  | Teamwork | Teamwork |  |
|  |  |  | Repitition of instructions/request to repeat information |  |  |
|  |  |  | Safe and efficient instument exchange |  |  |
|  |  |  | Safe and efficeint completion of surgeon requests |  |  |
|  |  |  | Assistant actions completed in correct and timely manner |  |  |
|  |  |  | Team actions completed inaccurately and in timely manner |  |  |
|  |  |  | Team complete requests accurately and in timely manner |  |  |
|  |  |  | Team react to surgeon requests in timely manner |  |  |
|  |  |  | Team confidence |  |  |
|  |  |  | Staff ability to move about OT |  |  |
|  |  |  | Team comfort in speaking up |  |  |
|  |  |  | Team attention |  |  |
|  |  |  | Leadership |  |  |
|  |  |  | Acknowledgement of team roles |  |  |
|  |  |  | Team preparedness |  |  |
|  | De-skilling (n=1) |  |  | De-skilling |  |
| **Organisation (n=173)** | RAS capital costs (n=21) | Purchase cost (n=5) | Captial investment/captial cost (base system, options of system, start-up reusable equipment) | Captial investment/captial cost (base system, options of system, start-up reusable equipment) |  |
|  |  |  | Cost of RAS system purchase/acquisition cost | Cost of RAS system purchase/acquisition cost | Cost of RAS system purchase/acquisition cost |
|  |  |  | Capital equipment cost |  |  |
|  |  |  | Capital costs |  |  |
|  |  |  | Purchase costs - where funds obtained, eg local charity |  |  |
|  |  | Equipment costs (n=12) | Equipment costs |  |  |
|  |  |  | Equipment and running costs based on number of procedures per year |  |  |
|  |  |  | Disposables | Disposables |  |
|  |  |  | Disposables cost |  |  |
|  |  |  | Disposable instruments cost |  |  |
|  |  |  | Cost of disposables |  |  |
|  |  |  | Disposable fees |  |  |
|  |  |  | Consumables | Consumables |  |
|  |  |  | Instruments and consumables cost |  |  |
|  |  |  | Reusables cost | Reusables cost |  |
|  |  |  | Cost of surgical implements |  |  |
|  |  |  | General surgery equipment lifespan | General surgery equipment lifespan |  |
|  |  | Maintenance and upgrade costs (n=4) | Service contract/maintenance cost | Service contract/maintenance cost | Service contract/maintenance cost |
|  |  |  | Cost of RAS system upgrades/renewal cost |  | Cost of RAS system upgrades/renewal cost |
|  |  |  | Clinical lifespan of computer hardware |  |  |
|  |  |  | Lifespan |  |  |
|  | Total cost of the operation (n=17) |  | Patient preparation (cost of) |  |  |
|  |  |  | Procedure costs |  |  |
|  |  |  | Operation cost |  |  |
|  |  |  | Operating cost |  |  |
|  |  |  | Attributable cost per procedure |  |  |
|  |  |  | Hospitalisation cost |  |  |
|  |  |  | Anaesthesia (cost of) | Anaesthesia (cost of) |  |
|  |  |  | General perioperative costs |  |  |
|  |  |  | Cost associated with perioperative AEs |  |  |
|  |  |  | Cost associated with postoperative care |  |  |
|  |  |  | Patient outcomes and costs up to 30 days post discharge |  |  |
|  |  |  | Cost of IV fluids |  |  |
|  |  |  | Cost of transfusion |  |  |
|  |  |  | Transfusion costs |  |  |
|  |  |  | Transfusion intra-operative |  |  |
|  |  |  | Transfusion post-operative |  |  |
|  |  |  | units of packed/transfused red blood cells (fewer for RAS) |  |  |
|  | Cost of sterilisation (n=8) |  | Incineration cost |  |  |
|  |  |  |  | decontamination on-site |  |
|  |  |  |  | Decontamination service |  |
|  |  |  |  | Specialist sterilisation |  |
|  |  |  | Risk of desterilisation |  |  |
|  |  |  | Cleaning (cost of) |  |  |
|  |  |  | Sterilisation (cost of) | Sterilisation (cost of) |  |
|  |  |  |  | Infection control |  |
|  | Post-discharge treatment costs (n=15) |  | Cost of cancer treatment |  |  |
|  |  |  | Adjuvant or salvage radiation |  |  |
|  |  |  | Radiotherapy cost (associated with positive surgical margins, includes radiation oncologist consult, physician assessments every six weeks and physician visit follow-up every three months after findings of positive surgical margins) |  |  |
|  |  |  |  | reduced rate of use of radiotherapy |  |
|  |  |  | Long-term healthcare cost in patients with prostate cancer (e.g. recurrence, secondary treatment) |  |  |
|  |  |  | Cost of medications |  |  |
|  |  |  | Pharmacy costs |  |  |
|  |  |  | Outpatient clinic costs |  |  |
|  |  |  | Diagnostic costs |  |  |
|  |  |  | Lab costs |  |  |
|  |  |  | PDE5 inhibitors cost (sildenafil) |  |  |
|  |  |  | Continence pads cost |  |  |
|  |  |  |  | Cost of stoma bags |  |
|  |  |  |  | Cost of ongoing care in the community |  |
|  |  |  | Outpatient cost |  |  |
|  | Overall economic/cost effectiveness (n=11) |  | Economic feasibility |  |  |
|  |  |  | QALYS | QALYS |  |
|  |  |  | Cost per QALY gained |  |  |
|  |  |  | ICER |  |  |
|  |  |  | Economic evaluation |  |  |
|  |  |  |  | Opportunity cost |  |
|  |  |  |  |  | Do other parts of the service suffer |
|  |  |  |  | Cost savings | Cost savings |
|  |  |  | Cost-effectiveness | Cost-effectiveness | Cost-effectiveness |
|  |  |  |  | Long-term financial evaluation |  |
|  |  |  |  | Utility |  |
|  | Overall efficiency (n=3) |  | Achieving Organisation goals |  |  |
|  |  |  |  | Organisational efficiency |  |
|  |  |  | Organisational feasibility |  |  |
|  | Volume/throughput (n=9) |  | Turnover time to next case | Turnover time to next case |  |
|  |  |  |  | Throughput |  |
|  |  |  | Number of patients treated/volume | Number of patients treated/volume | Number of patients treated/volume |
|  |  |  | 1-year hospital activity |  |  |
|  |  |  | Hospital volume | Hospital volume |  |
|  |  |  | Surgical volume |  |  |
|  |  |  | Cost by volume | Cost by volume |  |
|  |  |  |  | Capacity of the theatre |  |
|  |  |  |  |  | Number of robots available for general surgery |
|  | Standardisation of operative quality (n=6) |  | Access to tumour |  |  |
|  |  |  | Dual control/console |  |  |
|  |  |  |  | a second surgeon can take over the operation from another room or country |  |
|  |  |  | Disruption to flow of operation |  |  |
|  |  |  |  | Levels playing field/improves surgical standard/standardises surgery |  |
|  |  |  |  | Reduces variation in implant alignment |  |
|  | Impact on service delivery (n=2) |  | Retaining services |  |  |
|  |  |  | Integration into practice |  |  |
|  | Impact on infrastructure (n=15) |  | Having a dedicated or suitable operating room |  |  |
|  |  |  | Equivalent annual cost of total capital cost and hospital's overhead cost at 5 year service lifespan |  |  |
|  |  |  | Equivalent annual cost of total capital cost and hospital's overhead cost at 7 year service lifespan |  |  |
|  |  |  | Equivalent annual cost of total capital cost and hospital's overhead cost at 9 year service lifespan |  |  |
|  |  |  | Equivalent annual cost of total capital cost and hospital's overhead cost at 12 year service lifespan |  |  |
|  |  |  | Hospital overhead cost |  |  |
|  |  |  | Attributable cost per procedure |  |  |
|  |  |  | Cost to charge ratios (hospital to institution costs) |  |  |
|  |  |  | Administration (cost of) |  |  |
|  |  |  | Overhead and space (cost of) |  |  |
|  |  |  | Space |  |  |
|  |  |  | Theatre size | Theatre size |  |
|  |  |  |  |  | Cost of refiguring buildings |
|  |  |  | Connect smaller communities to larger academic centres |  |  |
|  |  |  |  | Expand to other services (e.g. ENT) |  |
|  | Impact on organisational reputation (n=1) |  |  | Organisational reputation |  |
|  | Organisational attitude (n=1) |  |  | Risk taking/risk evasive at organisational level |  |
|  | Impact on waiting list times (n=1) |  | Wait times | Wait times | Wait times |
|  | Equipment failure (n=14) |  | Equipment failure |  |  |
|  |  |  | Mechanical failure |  |  |
|  |  |  | Mechanical breakdown |  |  |
|  |  |  |  |  | Breakdowns |
|  |  |  | Instrument malfunction |  |  |
|  |  |  | Device failure rates | Device failure rates |  |
|  |  |  | System failure rates |  |  |
|  |  |  | Broken instrument tips |  |  |
|  |  |  | Failure of elecautery elements of intruments |  |  |
|  |  |  | Unrecoverable system faults |  |  |
|  |  |  | Conversions due to mechanical failure |  |  |
|  |  |  | Foot pedals not working |  |  |
|  |  |  | Camera issues |  |  |
|  |  |  | Computer navigation error |  |  |
|  | Workforce (n=49) | Staff fees/costs (n=5) | Wages (cost of) |  |  |
|  |  |  | Nursing fees |  |  |
|  |  |  | Surgeon professional fee |  |  |
|  |  |  | Other medical staff fees |  |  |
|  |  |  | Staff overtime |  |  |
|  |  | Impact on staff demands/roles (n=12) | Increased responsibility and demand for RAS nurses |  |  |
|  |  |  | New demands and challenges |  |  |
|  |  |  | Increased technical knowledge |  |  |
|  |  |  | Expanded surgical duties |  |  |
|  |  |  | Lack of role clarity - Nurses in one study described their increased responsibilities coinciding with a lack of clarity in the scope of their role during RS, suggesting a need for role clarity for the RS nurse |  |  |
|  |  |  | reduce travel costs (remote working) |  |  |
|  |  |  | Divison of labour and workload |  |  |
|  |  |  | Additional personnel requirements |  |  |
|  |  |  |  | Impact on support services |  |
|  |  |  | decentralise healthcare (remote working possible) |  |  |
|  |  |  |  | Re-model workforce (changed hours when operations can be performed) |  |
|  |  |  |  | Different models for delivering theatre teams |  |
|  |  | Training (n=17) | Clinician-led introduction of technology |  |  |
|  |  |  | Training. not only for surgeons, trainees but for surgical nurses, surgical residents, and anesthesia professionals | Training | Training |
|  |  |  | Training Low volume RAS patient cases which hampered surgical experience and skill in RS, especially among surgical residents |  |  |
|  |  |  | Cost of learning curve |  |  |
|  |  |  | credentialing process |  |  |
|  |  |  | Procedure specific training |  |  |
|  |  |  | Console training time |  |  |
|  |  |  | Live cases training |  |  |
|  |  |  | Residency training programmes |  |  |
|  |  |  | (no) standard training curriculum |  |  |
|  |  |  | (need for) international or national regulation of training and credentialling |  |  |
|  |  |  | Number of theatre personnel teams that require training - 2 to 4 depending on surgical volume |  |  |
|  |  |  | Training for hardware upates |  |  |
|  |  |  | Training for software updates |  |  |
|  |  |  | Training for central sterilsation supply dept staff |  |  |
|  |  |  | Opportunity for learning |  |  |
|  |  |  | Guidelines for nomination and overseeing of nomination of proctors for training |  |  |
|  |  | Staff skill/experience/support (n=4) | Having a surgical assistant experienced in RAS |  |  |
|  |  |  | Having a technical support rep onsite/available by telephone There is the possibility for technical issues to arise, and having a technical support representative available on-site or by phone to troubleshoot is key. The availability of technical support works to alleviate stress and improve confidence levels in case technical issues arise in the OR. One focus group study of perioperative nurses described the lack of a technical support system as creating concern among nurses about unexpected situations related to problems with machine errors |  |  |
|  |  |  | Skill mix |  |  |
|  |  |  | staff experience |  |  |
|  |  | Staff engagement (n=7) | Team leader - to coordinate and define roles, especially in the set-up phase, could improve efficiency, cooperation, and team trust and confidence during RS |  |  |
|  |  |  | Involving and engaging staff - Surgical staff discussed the importance of involving and engaging staff at multiple levels of the organization and creating a shared vision around RS systems, as opposed to the implementation of a RS system being surgeon-led |  |  |
|  |  |  | Board and staff-level support - This included ensuring there is board level and surgical staff support, the availability of comprehensive training, and ensuring theright skill set is available. Surgical staff viewed a RSS as allowing the hospital to be more competitive, in that it attracted patients and surgeons and was perceived as a mark of prestige |  |  |
|  |  |  |  | Alienate senior workforce |  |
|  |  |  |  | Theatre/ward staff involvement/education/engagement |  |
|  |  |  |  | Staff/nursing staff apprehension/unfamiliarity with new technology |  |
|  |  |  | Acceptability of technology |  |  |
|  |  | Staff motivation/attitude (n=2) | Staff privilege |  |  |
|  |  |  |  | Robot underused/enthusiasm change over time |  |
|  |  | Recruitment/retention (n=2) | improve recruitment and retention | improve recruitment and retention |  |
|  |  |  | Improve recruitment and retention in smaller communities through remote working |  |  |
| **Population (n=18)** | Impact on burden of illness (n=4) |  | Productivity/lost productivity up to 52 days post discharge |  |  |
|  |  |  | Return to work - cost | Return to work - cost |  |
|  |  |  | Return to normal activity - cost |  |  |
|  |  |  | Burden of illness |  |  |
|  | Caregiver burden (n=1) |  | Caregiver costs up to 52 days post discharge |  |  |
|  | Use of public money (n=2) |  |  | Use of public money/taxpayers' money/Diversion of resources from other treatments and/or overall healthcare fund |  |
|  |  |  |  |  | Tax payer point of view |
|  | Societal value (n=1) |  | Societal value |  |  |
|  | Equity of access (n=9) |  | Access/fair distribution of healthcare resources | Access/fair distribution of healthcare resources |  |
|  |  |  | Inequity between private and public healthcare | Inequity between private and public healthcare |  |
|  |  |  | Justice |  |  |
|  |  |  | Restricted patient selection and infringement on personal autonomy |  |  |
|  |  |  |  | Individual versus majority benefit |  |
|  |  |  |  | Split workforce/create competition between NHS boards and Trusts |  |
|  |  |  |  |  | Assistance to LMIC - surgeons can work in another country so could offer assistance to LMIC |
|  |  |  | Centralisation of services |  |  |
|  |  |  | Location of centre/geography | Location of centre/geography |  |
|  | Ethical value (n=1) |  | Ethical value |  |  |

**S5 Table – List of outcomes with their associated plain language definitions used for the Delphi**

| **How important is it that an evaluation of robotic assisted surgery** **can be shown to effect:** | | | |
| --- | --- | --- | --- |
| **#** | **Domain** | **Outcome** | **Definition** |
|  | *Patient outcomes* |  |  |
| **1** | Intra-operative complications (complications that may happen during the operation) | Procedure-specific injury | Any injury resulting from a specific surgical procedure, such as organ and/or nerve injury, and soft tissue damage during the operation |
| **2** |  | Blood loss/blood transfusion | Volume of blood lost during surgery and/or the number of units of blood put into a patient’s blood stream via a patient’s vein |
| **3** | Post-operative complications (complications that may happen after the operation) | Overall measure of complications (eg Clavien-Dindo – a system for measuring surgical complications) | Overall measure of any adverse event resulting from the operation |
| **4** |  | Infections | Any infection occurring after the operation |
| **5** |  | Wound related complications | An infection or injury at the wound site, such as a gap at the site of the wound that allows the organ to protrude through the muscle wall |
| **6** |  | Procedure-specific pain | Pain resulting from a specific surgical procedure (e.g. body site-specific pain) |
| **7** |  | Cognitive complications | Confusion or altered mental state as a result of the operation |
| **8** |  | Cerebrovascular complications | Any cerebrovascular complication, such as a temporary interruption in the blood supply to the brain, or stroke |
| **9** |  | Cardiovascular complications | Any cardiovascular complication, such as a heart attack, abnormal heart beat, high blood pressure, and blood clot |
| **10** |  | Renal complications | Any renal complication, such as dehydration, and when the kidneys stop working or don’t work as well as they should |
| **11** |  | Pulmonary/respiratory complications | Any pulmonary/respiratory complication, such as a lung infection, a problem with the valve between the heart and the pulmonary artery that allows blood to flow back into the heart, and a blocked blood vessel in the lungs |
| **12** |  | Urinary complications | Any urinary complication, such as incontinence, being unable to empty all the urine from the bladder, urine leaking into the body, and scarring or narrowing of the urinary tract |
| **13** |  | Uterovaginal complications | Any uterovaginal complication, such as fluid under the skin, vaginal leak or discharge and an abnormal passageway or opening between the urinary tube and the vagina |
| **14** |  | Gastrointestinal complications | Any gastrointestinal complication, such as pain and/or swelling of the gallbladder/gallstones, and blockage of the small intestine |
| **15** |  | Head and neck complications | Any head and neck complication, such as leaking saliva outside the throat, difficulty understanding or producing words, and temporary rough or harsh sounding voice |
| **16** |  | Urological complications | Any urological complication, such as swelling of the tube behind the testicles |
| **17** |  | Endocrine complications (complications with the glands that produce hormones that help control the way the body works, including metabolism, mood and sleep. | Any endocrine complication, such as the level of calcium in the blood dropping too low), or changes to the level of hormone that regulates calcium in the body |
| **18** |  | Functional complications | Complications resulting in difficulties performing tasks or activities, such as ( problems having sex after an operation to remove the prostate – a gland that helps make semen) |
| **19** | Length of ICU stay |  | Length of time spent in ICU |
| **20** | Length of hospital stay |  | Length of time spent in the hospital from admission to discharge |
| **21** | Treatment effectiveness | Operative progress | The measure of success of the immediate success of the procedure, including care given after the procedure while the patient is in hospital, such as duration of catherisation, in-house analgesia usage, and drain duration |
| **22** |  | Overall measure of treatment effectiveness/benefit | How successful the procedure was |
| **23** | Quality of life | Overall quality of life | Overall state of the patient’s physical and mental wellbeing |
| **24** | Quality of life | Disease-specific quality of life | How well the patient feels physically and emotionally in relation to their specific |
| **25** | Time to recovery | Time to physical recovery | Time to return to activities such as walking, running, swimming, cycling, physical labour, climbing stairs, gardening etc |
| **26** |  | Time to role recovery | Length of time off work after the operation in days |
| **27** | Patient perception of their treatment/operation |  | What patients think, feel and understand about their operation, including any safety/trust concerns, |
| **28** | Patient experience of their treatment/operation | Emotion | Patient mental health, apprehension and psychosocial impact of surgery |
| **29** |  | Patient-surgeon-nurse relationship | Nature and quality of communication with surgeons and nurses, including trust in surgeon, relationship with surgeon and quality of informed consent |
| **30** |  | Cosmesis | The extent to which patient is content with the cosmetic results of surgery |
| **31** |  | Overall satisfaction | The degree to which all expectations or needs have been fulfilled |
| **32** |  | Satisfaction with procedure-specific outcome | The degree to which expectations of the specific operative procedure have been fulfilled |
| **33** | Readmission | Post-operative readmission/reoperation | Any subsequent admission to hospital and/or subsequent operation |
| **34** | Disease progression/treatment failure |  | Worsening or persistent signs or symptoms of disease after the disease has been treated |
| **35** | Subsequent treatment |  | Impact on patient of subsequent treatment, such as adjuvant radiation, outpatient clinic |
| **36** | Mortality |  | Death from any cause |
| **37** | Treatment affordability |  | How financially affordable the operation is for the patient (this can vary between different countries and health care systems) |
|  | *Surgeon outcomes* |  |  |
| **38** | Learning curve |  | The time taken and/or the number of procedures required for surgeon to perform a procedure independently with a reasonable outcome |
| **39** | Surgeon Motivation |  | The surgeon’s willingness or desire to perform or learn new skills or adopt new technology |
| **40** | Operative control | Control of instruments | How well the surgeon can handle surgical instruments |
| **41** |  | Visualisation | The field of vision available to the surgeon during the procedure |
| **42** |  | Precision/accuracy | The surgeon’s ability to carry out the procedure accurately without error |
| **43** |  | Surgeon autonomy | The surgeon’s independent control over the procedure and surrounding environment |
| **44** | Operative time |  | Time taken for the surgeon to perform the operation |
| **45** | Physical impact/surgeon ergonomics | Comfort/physical strain | The degree of physical comfort/discomfort felt by the surgeon during the procedure, such as neck, shoulder, upper back, and hand strain |
| **46** |  | Fatigue | Fatigue experienced by the surgeon during the procedure |
| **47** |  | Nausea/vertigo | The surgeon experiences feelings of sickness or dizziness during the operative procedure |
| **48** |  | Impact on length of surgeon's working life | How long a surgeon is able to physically perform a procedure |
| **49** | Emotional/cognitive impact | Emotional impact | Stress experienced by the surgeon or surgeon’s ability to disconnect emotionally from the patient during the procedure |
| **50** |  | Cognitive impact | The quality of the surgeon’s ability to make decisions |
| **51** |  | Impact on Concentration | The surgeon’s ability to focus or become distracted during the procedure |
| **52** | Situational awareness |  | The surgeon’s awareness of the dynamics of the surrounding environment during the procedure |
| **53** | Surgeon satisfaction |  | The surgeon’s overall satisfaction with the procedure |
| **54** | Quality of Communication in operative team |  | The quality and ease of communication between the surgeon or surgeons and the rest of the surgical team |
| **55** | De-skilling |  | The loss of skills in a given surgical technique or procedure |
|  | *Organisational outcomes* |  |  |
| **56** | Capital costs of operative equipment | Purchase cost | The total cost of purchasing the operative equipment |
| **57** |  | Equipment costs | The cost of purchasing consumables and disposables |
| **58** |  | Maintenance and upgrade costs | The cost of maintaining and upgrading the operative equipment |
| **59** | Total cost of the operation |  | The total cost of the operation |
| **60** | Cost of sterilisation of equipment |  | Cost of specialist sterilisation and decontamination procedures |
| **61** | Post-discharge treatment costs |  | The cost of health care in the community such as continence pads, stoma bags etc |
| **62** | Overall economic/cost-effectiveness |  | The value for money provided by a service, such as the cost-effectiveness of treatment route (medical management or surgery), calculated by dividing cost by success rate (defined by the quality of life after treatment) |
| **63** | Overall Efficiency |  | Overall efficiency/stream-lining of services |
| **64** | Volume/throughput |  | The number of patients that can be treated and the turnover time to the next case |
| **65** | Standardisation of operative quality |  | The degree to which variation in a given procedure is reduced and/or equal outcomes are achieved |
| **66** | Impact on service delivery |  | The way a new service is set up and delivered, and how existing services are altered and adapted |
| **67** | Impact on infrastructure |  | The impact on basic physical and organizational structures and facilities, such as building management, power supplies, IT systems, hardware, software, and people |
| **68** | Impact on organisational reputation |  | How the organisation is perceived by others |
| **69** | Organisational attitude |  | The vision and ambition of the organisation, such as the organisation’s attitude to innovation and new technology |
| **70** | Equipment failure |  | Any equipment failure |
| **71** | Impact on waiting list times |  | The time it takes for a patient to receive treatment after being referred to hospital |
| **72** | Impact on workforce | Impact on Staff fees/costs | Any staff fees or costs, including salary costs |
| **73** |  | Impact on staff demands/roles | Any impact on individual staff and their roles as a result of a change in service |
| **74** |  | Impact on Training | Cost and feasibility of staff training |
| **75** |  | Staff skill/experience/support required | Levels of skilled or experiences staff and support required for the operative procedure |
| **76** |  | Staff engagement with operative approach | The level to which staff are committed to the operative approach, or conversely levels of apprehension or alienation |
| **77** |  | Staff motivation/attitude | Staff motivation to deliver the operative approach |
| **78** |  | Recruitment/retention | Ability to recruit new staff and/or retain existing staff to deliver the operative approach |
|  | *Population level outcomes* |  |  |
| **79** | Impact on burden of illness |  | The impact of the operative approach on the levels of disease/management of a specific condition in the population |
| **80** | Caregiver burden |  | Impact of the operative approach on care givers |
| **81** | Use of public money |  | Impact on the use of money raised from public taxation or public assets |
| **82** | Societal value |  | Extent to which the operative approach is in keeping with societal values, such as fairness and dignity |
| **83** | Ethical value |  | Extent to which the operative approach is in keeping with ethical values, such as health maximisation, avoidance of harm, acting for the benefit of others |
| **84** | Environmental/carbon footprint |  | The environmental impact and carbon footprint associated with the procedure |
| **85** | Equity of access |  | Impact on the degree to which people have equal access to a given treatment or procedure |

**S6 File – Organisations that circulated the Delphi**

| **Patient Organisations – directly to organisation generally or via known contact and/or PPI lead*** | **Coverage** |
| --- | --- |
| Bowel cancer UK | UK |
| National Cancer Research Institute | UK |
| Cochrane Consumer Network | UK |
| The European Patients’ Academy on Therapeutic Innovation (EUPATI) | Europe |
| National Institute for Health Research (NIHR) Surgical MedTech Co-operative | UK |
| NIHR Cambridge Biomedical Research Centre Patient and Public Involvement Team | Cambridge, UK |
| Health Technology Assessment international (HTAi) Patient and Citizen Involvement interest group | International |
| Association of Medical Research Charities (AMRC) | UK |
| EUORDIS | Europe |
| International Alliance of Patient Organisations | International |
| **‘Researcher’ facing organisations** |  |
| AO Spine Research Objectives and Common Data Elements for Degenerative Cervical Myelopathy (AO Spine RECODE-DCM) | International |
| **Surgical organisations** |  |
| COVID surgery global health group | International |
| Association of Surgeons of Great Britain and Ireland (ASGBI) | UK and Ireland |
| Association of Laparoscopic Surgeons of Great Britain and Ireland (ALSGBI) | UK and Ireland |
| **Social Media** | |
| Facebook: @UoAsmmsn | International |
| Twitter: @hsru_aberdeen | International |
| Linkedin | International |

**S7 File – Consensus Meeting Documents**

**Robotic core outcome sets (RoboCOS): What outcomes are important for evaluating robotic assisted surgery as a service level change?**

**Information Sheet**

This study is interested in exploring a range of people’s views about the introduction and scale-up of robot-assisted surgical services in the health service. The people we would like to include are those who are involved in the set-up and delivery of robot-assisted surgical services and those who receive it. You are being invited to a meeting to reach agreement on what core outcomes should be considered when testing robot-assisted surgery in the health service. The meeting will take place on 22^nd^ July on Zoom.

When we do research to test how effective and efficient robotic assisted surgery is, we want to look at the outcomes of that process. At the moment, different studies looking at different ways of testing robotic assisted surgery often measure different outcomes. Imagine there are two studies that test the performance of robotic assisted surgery for the removal of the prostate gland (a procedure called prostatectomy) in people with prostate cancer.

- Study A - researchers measure positive surgical margins (whether there are any cancer cells present in the edges of the tissue removed during surgery) as an outcome
- Study B – researchers measure the number of people without cancer recurrence at five years as an outcome

When the two studies are finished, their results cannot be compared or combined because they used different outcomes. To produce meaningful results when bringing results of different studies together, we need the outcomes to be the same across studies. Similarly, there might be outcomes that have not been considered in research studies but are important to different groups of people. We can solve this problem by getting agreement from relevant communities on what main outcomes should be considered as core when evaluating robotic assisted surgery. These main outcomes are a minimum core set known as a ‘core outcome set’ and need to be relevant to and endorsed by patients, researchers, clinicians, hospital service managers, and policy makers.

There are several steps involved in the development of a core outcome set. First to identify what outcomes have been reported previously, then to ask the relevant groups (in this case patients and members of the public, surgeons and other clinical staff, hospital service managers, policy makers, and researchers) what outcomes they consider to be important, and then to get agreement on what the core set should contain. It is this final agreement on what the core set should contain that the meeting on 22^nd^ July will focus on. You will be presented with each of the outcomes and asked to vote on whether they are considered important enough to be considered ‘core’ for the evaluation of robotic assisted surgery.

We will go over the aim of the project and the process again on the day. If you would like to participate in the consensus meeting, or if you have any questions about taking part, please contact Clare Robertson by emailing [c.robertson@abdn.ac.uk](mailto:c.robertson@abdn.ac.uk)

**Consensus meeting for the development of a core outcome set for the evaluation of robotic assisted surgery as a service level change (RoboCOS)**

**10am to 3pm UK time**

**22^nd^ July**

Join Zoom Meeting

<https://us02web.zoom.us/j/88271311568?pwd=SjRkZmZmTUdaQjEwWXdienE2MjNSZz09>

Meeting ID: 882 7131 1568

Passcode: 080428

**Agenda**

| 10:00 | Welcome and brief introductions |  |
| --- | --- | --- |
| 10:15 | Outline of the RoboCOS study: progress to date and plans for the day |  |
| 10:45 | Presentation of outcomes |  |
| 11:00 | Discussion of outcomes with agreement |  |
| 12:00 | Lunch |  |
| 12:30 | Voting on outcomes with no consensus |  |
| 14:30 | Summary and next steps |  |

**Summary scores from Delphi Round 2**

The following four pages present the summary results from our online survey that asked people like you to score how important they thought these outcomes were in terms of whether they should be included in a core outcome set for the service-level assessment of robotic assisted surgery.

In the survey each outcome was presented and people were asked to rate the importance on a scale from 1-9, with 1 being not important and 9 being very important. When we analysed the results we grouped the responses into the following categories:

- 1-3: not important
- 4-6: unsure
- 7-9: very important

We had previously agreed that any outcomes considered important by more than 90% of the group for patient-level outcomes, and 70% of the group for all other outcomes would be taken forward into the core set. Any outcomes where the majority of the responders (i.e. 70% or less of the group for patient-level outcomes and 50% or less of the group for other outcomes) did not consider the outcome important enough for inclusion in the core set would not be considered further. The other outcomes are the ones for which agreement could not be reached and require further discussion.

The tables below present the overall group scores for each outcome. At the meeting we will spend most time talking about the outcomes for which no consensus was reached (Tables 1 to 4) but we would also like to confirm those which were considered of highest importance or ‘core’(Tables 5 to 8) and agree that those for which the majority did not think were important can be excluded (Table 9).

**How to interpret the data presented in the tables.**


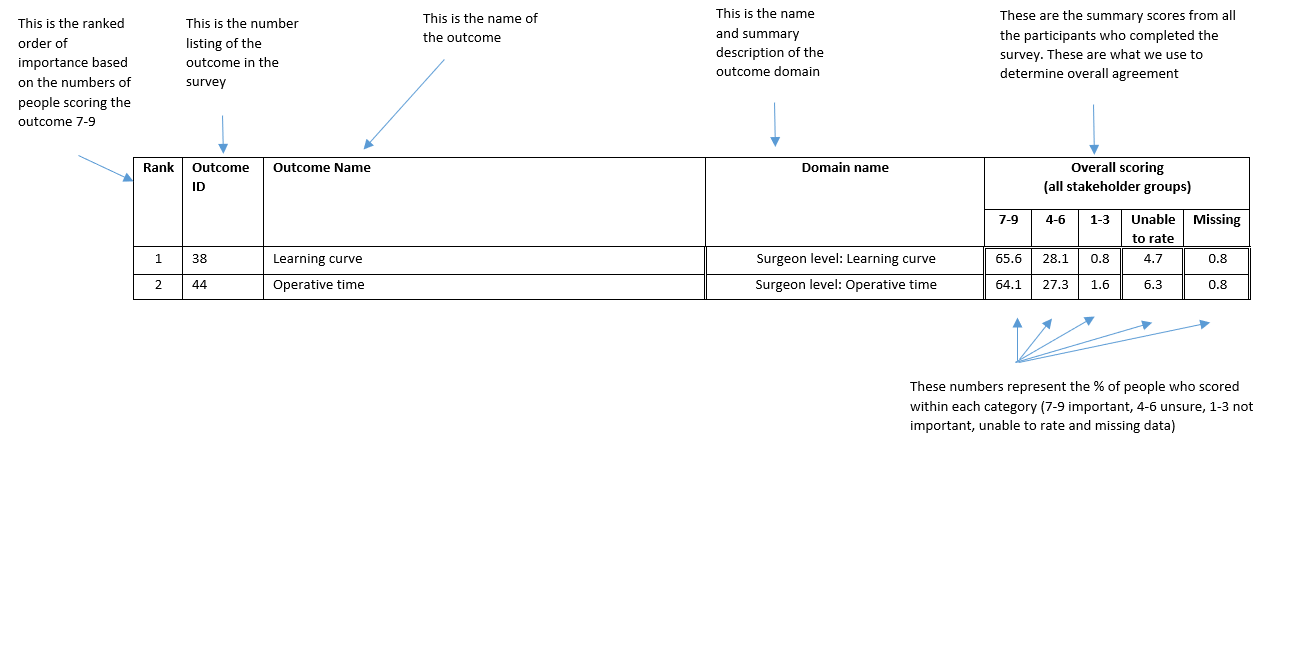


**Legend:**

|  | **Proposed not for discussion - achieved ‘consensus in’ during Delphi survey** |
| --- | --- |
|  | **For discussion – Scored highly during Delphi but just outside of ‘consensus in’ criteria. Highly ranked but not the top** |
|  | **For discussion – Overall ‘consensus out’ but high disagreement between groups where one group scored the outcome very differently to others.** |
|  | **Proposed not for discussion – Achieved ‘consensus out’ criteria following the Delphi survey** |

**S8 All Tables**

**Tables 1-4 Outcomes reaching consensus for inclusion in the final core outcome set**

**Table 1 Patient level outcomes reaching consensus for inclusion in the final core outcome set**

| **Rank** | **Outcome ID** | **Outcome Name** | **Domain name** | **Overall scoring**  **(all stakeholder groups)** | | | | |
| --- | --- | --- | --- | --- | --- | --- | --- | --- |
|  |  |  |  | **7-9** | **4-6** | **1-3** | **Unable to rate** | **Missing** |
| 1 | 24 | Disease-specific quality of life | Patient level: Quality of life | 93.0 | 4.7 |  | 2.3 |  |
| 2 | 36 | Mortality | Patient level: Mortality | 93.0 | 3.9 | 0.8 | 1.6 | 0.8 |
| 3 | 1 | Procedure-specific injury | Patient level: Intra-operative complications (complications that may happen during the operation) | 93.0 | 3.9 |  | 3.1 |  |
| 4 | 23 | Overall quality of life | Patient level: Quality of life | 92.2 | 5.5 |  | 2.3 |  |
| 5 | 22 | Overall measure of treatment effectiveness/benefit | Patient level: Treatment effectiveness | 91.4 | 6.3 |  | 2.3 |  |

**Table 2 Surgeon level outcomes reaching consensus for inclusion in the final core outcomes set**

| **Rank** | **Outcome ID** | **Outcome Name** | **Domain name** | **Overall scoring**  **(all stakeholder groups)** | | | | |
| --- | --- | --- | --- | --- | --- | --- | --- | --- |
|  |  |  |  | **7-9** | **4-6** | **1-3** | **Unable to rate** | **Missing** |
| 1 | 42 | Precision/accuracy | Surgeon level: Operative control | 84.4 | 7.8 | 1.6 | 5.5 | 0.8 |
| 2 | 41 | Visualisation | Surgeon level: Operative control | 76.6 | 14.1 | 2.3 | 6.3 | 0.8 |
| 3 | 40 | Control of instruments | Surgeon level: Operative control | 75.8 | 14.8 | 3.1 | 5.5 | 0.8 |
| 4 | 43 | Surgeon autonomy | Surgeon level: Operative control | 71.9 | 18.8 | 2.3 | 6.3 | 0.8 |

**Table 3 Organisation level outcomes reaching consensus for inclusion in the final core outcome set**

| **Rank** | **Outcome ID** | **Outcome Name** | **Domain name** | **Overall scoring**  **(all stakeholder groups)** | | | | |
| --- | --- | --- | --- | --- | --- | --- | --- | --- |
|  |  |  |  | **7-9** | **4-6** | **1-3** | **Unable to rate** | **Missing** |
| 1 | 70 | Equipment failure | Organisation level: Equipment failure | 78.1 | 15.6 | 2.3 | 3.1 | 0.8 |
| 2 | 65 | Standardisation of operative quality | Organisation level: Standardisation of operative quality | 72.7 | 18.0 | 1.6 | 7.0 | 0.8 |

**Table 4 Population level outcomes reaching consensus for inclusion in the final core outcome set**

| **Rank** | **Outcome ID** | **Outcome Name** | **Domain name** | **Overall scoring**  **(all stakeholder groups)** | | | | |
| --- | --- | --- | --- | --- | --- | --- | --- | --- |
|  |  |  |  | **7-9** | **4-6** | **1-3** | **Unable to rate** | **Missing** |
|  | 85 | Equity of access | Population level: Equity of access | 77.3 | 18.8 | 1.6 | 1.6 | 0.8 |

**Tables 5-8 Outcomes with no consensus**

**Table 5 Patient outcomes with no consensus**

| **Rank** | **Outcome ID** | **Outcome Name** | **Domain name** | **Overall scoring**  **(all stakeholder groups)** | | | | |
| --- | --- | --- | --- | --- | --- | --- | --- | --- |
|  |  |  |  | **7-9** | **4-6** | **1-3** | **Unable to rate** | **Missing** |
| 1 | 3 | Overall measure of complications (eg Clavien-Dindo– a system for measuring surgical complications) | Patient level: Intra-operative complications (complications that may happen during the operation | 85.9 | 6.3 | 1.6 | 6.3 |  |
| 2 | 25 | Time to physical recovery | Patient level: Time to recovery | 84.4 | 13.3 | 0.8 | 1.6 |  |
| 3 | 33 | Post-operative readmission/reoperation | Patient level: Readmission | 84.4 | 11.7 | 1.6 | 1.6 | 0.8 |
| 4 | 34 | Outcomes post-hospital: Disease progression/treatment failure | Patient level: Disease progression/treatment failure | 88.3 | 8.6 |  | 2.3 | 0.8 |
| 5 | 32 | Satisfaction with procedure-specific outcome | Patient level: Patient perception of their treatment/operation | 78.1 | 19.5 | 0.8 | 1.6 |  |
| 6 | 21 | Operative progress | Patient level: Treatment effectiveness | 78.1 | 18.0 | 0.8 | 3.1 |  |
| 7 | 35 | Outcomes post-hospital: Subsequent treatment | Patient level: Subsequent treatment | 75.8 | 21.9 |  | 1.6 | 0.8 |
| 8 | 31 | Overall satisfaction | Patient level: Patient perception of their treatment/operation | 74.2 | 22.7 | 1.6 | 1.6 |  |
| 9 | 26 | Time to role recovery | Patient level: Time to recovery | 74.2 | 22.7 | 0.8 | 2.3 |  |
| 10 | 11 | Pulmonary/respiratory complications | Patient level: Post-operative complications (complications that may happen after the operation) | 74.2 | 17.2 | 3.9 | 4.7 |  |
| 11 | 4 | Infections | Patient level: Post-operative complications (complications that may happen after the operation) | 77.3 | 19.5 | 0.8 | 2.3 |  |
| 12 | 27 | Patient perception of their treatment/operation | Patient level: Patient perception of their treatment/operation | 70.3 | 25.8 | 1.6 | 2.3 |  |
| 13 | 2 | Blood loss/blood transfusion | Patient level: Intra-operative complications (complications that may happen during the operation) | 70.3 | 25.8 | 0.8 | 3.1 |  |
| 14 | 9 | Cardiovascular complications | Patient level: Post-operative complications (complications that may happen after the operation) | 70.3 | 23.4 | 2.3 | 3.9 |  |

**Table 6 Surgeon outcomes with no consensus**

| **Rank** | **Outcome ID** | **Outcome Name** | **Domain name** | **Overall scoring**  **(all stakeholder groups)** | | | | |
| --- | --- | --- | --- | --- | --- | --- | --- | --- |
|  |  |  |  | **7-9** | **4-6** | **1-3** | **Unable to rate** | **Missing** |
| 1 | 38 | Learning curve | Surgeon level: Learning curve | 65.6 | 28.1 | 0.8 | 4.7 | 0.8 |
| 2 | 44 | Operative time | Surgeon level: Operative time | 64.1 | 27.3 | 1.6 | 6.3 | 0.8 |
| 3 | 51 | Impact on Concentration (surgeon) | Surgeon level: Emotional/cognitive impact | 62.5 | 26.6 | 3.9 | 6.3 | 0.8 |
| 4 | 45 | Comfort/physical strain (surgeon) | Surgeon level: Physical impact/surgeon ergonomics | 57.0 | 35.2 | 1.6 | 5.5 | 0.8 |
| 5 | 46 | Fatigue (surgeon) | Surgeon level: Physical impact/surgeon ergonomics | 57.0 | 35.2 | 1.6 | 5.5 | 0.8 |
| 6 | 48 | Impact on length of surgeon's working life | Surgeon level: Physical impact/surgeon ergonomics | 52.3 | 38.3 | 2.3 | 6.3 | 0.8 |
| 7 | 53 | Surgeon satisfaction | Surgeon level: Emotional/cognitive impact | 54.7 | 32.8 | 3.9 | 7.8 | 0.8 |
| 8 | 54 | Quality of Communication in operative team | Surgeon level: Quality of Communication in operative team | 56.3 | 35.2 | 3.1 | 4.7 | 0.8 |

**Table 7 Organisation level outcomes with no consensus**

| **Rank** | **Outcome ID** | **Outcome Name** | **Domain name** | **Overall scoring**  **(all stakeholder groups)** | | | | |
| --- | --- | --- | --- | --- | --- | --- | --- | --- |
|  |  |  |  | **7-9** | **4-6** | **1-3** | **Unable to rate** | **Missing** |
| 1 | 63 | Overall Efficiency | Organisation level: Overall Efficiency | 64.8 | 27.3 | 3.1 | 3.9 | 0.8 |
| 2 | 62 | Overall economic/cost-effectiveness | Organisation level: Overall economic/cost-effectiveness | 63.3 | 30.5 |  | 5.5 | 0.8 |
| 3 | 71 | Impact on waiting list times | Organisation level: Impact on waiting list times | 54.7 | 39.8 | 2.3 | 2.3 | 0.8 |
| 4 | 74 | Impact on Training | Organisation level: Impact on workforce | 54.7 | 36.7 | 3.9 | 3.9 | 0.8 |
| 5 | 58 | Maintenance and upgrade costs | Organisation level: Capital costs of operative equipment | 54.7 | 36.7 | 3.1 | 4.7 | 0.8 |
| 6 | 64 | Volume/throughput | Organisation level: Volume/throughput | 54.7 | 35.2 | 2.3 | 7.0 | 0.8 |
| 7 | 59 | Total cost of the operation | Organisation level: Total cost of the operation | 53.9 | 38.3 | 1.6 | 5.5 | 0.8 |

**Table 8 Population outcomes with no consensus**

| **Rank** | **Outcome ID** | **Outcome Name** | **Domain name** | **Overall scoring**  **(all stakeholder groups)** | | | | |
| --- | --- | --- | --- | --- | --- | --- | --- | --- |
|  |  |  |  | **7-9** | **4-6** | **1-3** | **Unable to rate** | **Missing** |
| 1 | 79 | Impact on burden of illness | Population level: Impact on burden of illness | 53.1 | 40.6 | 2.3 | 3.1 | 0.8 |
| 2 | 81 | Use of public money | Population level: Use of public money | 52.3 | 39.8 | 5.5 | 1.6 | 0.8 |

**Table 9 Outcomes reaching consensus for exclusion from the final core outcome set**

| **Rank** | **Outcome ID** | **Outcome Name** | **Domain name** | **Overall scoring**  **(all stakeholder groups)** | | | | |
| --- | --- | --- | --- | --- | --- | --- | --- | --- |
|  |  |  |  | **7-9** | **4-6** | **1-3** | **Unable to rate** | **Missing** |
| Patient outcomes | | | | | | | | |
| 1 | 5 | Wound related complications | Patient level: Post-operative complications (complications that may happen after the operation) | 69.5 | 25.0 | 3.1 | 2.3 |  |
| 2 | 14 | Gastrointestinal complications | Patient level: Post-operative complications (complications that may happen after the operation) | 69.5 | 21.1 | 4.7 | 4.7 |  |
| 3 | 10 | Renal complications | Patient level: Post-operative complications (complications that may happen after the operation) | 68.8 | 21.1 | 5.5 | 4.7 |  |
| 4 | 6 | Procedure-specific pain | Patient level: Post-operative complications (complications that may happen after the operation) | 68.0 | 27.3 | 2.3 | 2.3 |  |
| 5 | 8 | Cerebrovascular complications | Patient level: Post-operative complications (complications that may happen after the operation) | 66.4 | 25.0 | 3.9 | 4.7 |  |
| 6 | 18 | Functional complications | Patient level: Post-operative complications (complications that may happen after the operation) | 64.1 | 26.6 | 3.9 | 5.5 |  |
| 7 | 19 | Length of ICU stay | Patient level: Length of ICU stay | 63.3 | 32.0 | 3.1 | 1.6 |  |
| 8 | 7 | Cognitive complications | Patient level: Post-operative complications (complications that may happen after the operation) | 63.3 | 28.1 | 4.7 | 3.9 |  |
| 9 | 12 | Urinary complications | Patient level: Post-operative complications (complications that may happen after the operation) | 62.5 | 27.3 | 5.5 | 4.7 |  |
| 10 | 20 | Length of hospital stay | Patient level: Length of hospital stay | 60.9 | 34.4 | 3.1 | 1.6 |  |
| 11 | 13 | Uterovaginal complications | Patient level: Post-operative complications (complications that may happen after the operation) | 60.2 | 28.9 | 6.3 | 4.7 |  |
| 12 | 16 | Urological complications | Patient level: Post-operative complications (complications that may happen after the operation) | 57.8 | 30.5 | 5.5 | 6.3 |  |
| 13 | 37 | Treatment affordability | Patient level: Treatment affordability | 53.9 | 39.8 | 1.6 | 3.9 | 0.8 |
| 14 | 15 | Head and neck complications | Patient level: Post-operative complications (complications that may happen after the operation) | 52.3 | 35.2 | 7.8 | 4.7 |  |
| 15 | 17 | Endocrine complications | Patient level: Post-operative complications (complications that may happen after the operation) | 48.4 | 36.7 | 7.8 | 7.0 |  |
| 16 | 28 | Emotion | Patient level: Patient perception of their treatment/operation | 40.6 | 50.8 | 6.3 | 2.3 |  |
| 17 | 29 | Patient-surgeon-nurse relationship | Patient level: Patient perception of their treatment/operation | 37.5 | 53.1 | 7.8 | 1.6 |  |
| 18 | 30 | Cosmesis | Patient level: Patient perception of their treatment/operation | 32.0 | 52.3 | 8.6 | 7.0 |  |
| Surgeon outcomes | | | | | | | | |
| 1 | 47 | Nausea/vertigo | Surgeon level: Physical impact/surgeon ergonomics | 48.4 | 38.3 | 5.5 | 7.0 | 0.8 |
| 2 | 52 | Situational awareness | Surgeon level: Situational awareness | 47.7 | 38.3 | 3.9 | 9.4 | 0.8 |
| 3 | 50 | Cognitive impact | Surgeon level: Emotional/cognitive impact | 42.2 | 43.0 | 6.3 | 7.8 | 0.8 |
| 4 | 39 | Surgeon Motivation | Surgeon level: Surgeon Motivation | 40.6 | 44.5 | 7.8 | 6.3 | 0.8 |
| 5 | 49 | Emotional impact | Surgeon level: Emotional/cognitive impact | 33.6 | 50.8 | 7.0 | 7.8 | 0.8 |
| 6 | 55 | De-skilling | Surgeon level: De-skilling | 29.7 | 55.5 | 3.9 | 10.2 | 0.8 |
| Organisation outcomes | | | | | | | | |
| 1 | 57 | Equipment costs | Organisation level: Capital costs of operative equipment | 49.2 | 43.0 | 2.3 | 4.7 | 0.8 |
| 2 | 78 | Recruitment/retention | Organisation level: Impact on workforce | 49.2 | 39.1 | 6.3 | 4.7 | 0.8 |
| 3 | 75 | Staff skill/experience/support required | Organisation level: Impact on workforce | 48.4 | 43.0 | 3.9 | 3.9 | 0.8 |
| 4 | 77 | Staff motivation/attitude | Organisation level: Impact on workforce | 48.4 | 41.4 | 5.5 | 3.9 | 0.8 |
| 5 | 56 | Purchase cost | Organisation level: Capital costs of operative equipment | 46.1 | 44.5 | 3.9 | 4.7 | 0.8 |
| 6 | 76 | Staff engagement with operative approach | Organisation level: Impact on workforce | 43.0 | 47.7 | 4.7 | 3.9 | 0.8 |
| 7 | 61 | Post-discharge treatment costs | Organisation level: Post-discharge treatment costs | 42.2 | 48.4 | 3.1 | 5.5 | 0.8 |
| 8 | 60 | Cost of sterilisation of equipment | Organisation level: Cost of sterilisation of equipment | 42.2 | 44.5 | 7.0 | 5.5 | 0.8 |
| 9 | 66 | Impact on service delivery | Organisation level: Impact on service delivery | 38.3 | 56.3 | 0.8 | 3.9 | 0.8 |
| 10 | 72 | Impact on Staff fees/costs | Organisation level: Impact on workforce | 32.8 | 55.5 | 6.3 | 4.7 | 0.8 |
| 11 | 73 | Impact on staff demands/roles | Organisation level: Impact on workforce | 32.0 | 57.8 | 4.7 | 4.7 | 0.8 |
| 12 | 67 | Impact on infrastructure | Organisation level: Impact on infrastructure | 30.5 | 62.5 | 2.3 | 3.9 | 0.8 |
| 13 | 69 | Organisational attitude | Organisation level: Organisational attitude | 28.1 | 53.1 | 14.1 | 3.9 | 0.8 |
| 14 | 68 | Impact on organisational reputation | Organisation level: Impact on organisational reputation | 27.3 | 52.3 | 15.6 | 3.9 | 0.8 |
| Population outcomes | | | | | | | | |
| 1 | 82 | Societal value | Population level: Societal value | 46.1 | 48.4 | 1.6 | 3.1 | 0.8 |
| 2 | 84 | Environmental/carbon footprint | Population level: Societal value | 43.8 | 42.2 | 9.4 | 3.9 | 0.8 |
| 3 | 83 | Ethical value | Population level: Ethical value | 43.0 | 49.2 | 3.1 | 3.9 | 0.8 |
| 4 | 80 | Caregiver burden | Population level: Caregiver burden | 37.5 | 51.6 | 5.5 | 4.7 | 0.8 |

**S9 File - Consensus Meeting Detailed Methods**

The consensus meeting was chaired by a trials methodologist who was part of the study team (MC) with expertise on COS development methodology and consensus facilitation. Outcomes that had reached consensus ‘in’ across the whole group from the Delphi R2 were presented first, followed by outcomes that reached consensus ‘out’ across the whole group from R2. Participants were asked to confirm they agreed (or not) with the inclusion or exclusion of these outcomes in the COS.

Outcomes scored as ‘no consensus’ for which 75% of all groups had scored 7-9 for patient level outcomes and 60% of all groups had scored 7-9 for all other outcomes (n=12), and as such have more potential to be included in the COS were voted by the group. See supplementary information file S9 Table for a summary of these pre-determined re-scoring rules.

Views for and against inclusion of the outcomes for which there was ‘no consensus’ after R2 were sought by the meeting chair. Discussion was supplemented with the following questions: Is this outcome currently covered in the COS (i.e. is it a surrogate for something already in)?; Does the group think it is really important to add?; Should it replace an outcome that is already in (i.e. is there an outcome in the COS that is a surrogate for this)? . Following discussion, participants were invited to vote on each outcome anonymously on using polls set up within Zoom. Following voting of each outcome, the results were presented to participants at the whole group results. The consensus definition used in the Delphi survey (see consensus definition section above) was applied to the result to determine consensus ‘in’ or ‘out’ for the outcome to be included in the final COS. Participants considered outcomes that achieved consensus ‘in’ after R2 and voted on any outcomes that were considered to have overlap in the final COS. Agreement of a pre-determined threshold of 70% or more had to be reached to remove any of these outcomes from the final COS.

**S10 Table - Rules for determining voting on outcomes during consensus meeting**

| **Outcome groups** | **Rules for voting** |
| --- | --- |
| Consensus in  n=12 | As per the predefined consensus criteria, outcomes with more than 90% or more participants scoring 7-9 for patient outcomes and more than 70% for all other outcomes were considered ‘in’. It was proposed to consensus meeting participants that these outcomes should not be re-voted unless another outcome voted ‘in’ had significant overlap. This was agreed. Five outcomes were put forward for re-voting |
| Consensus out  n=61 | As per the predefined consensus criteria, outcomes with fewer than 75% of participants scoring 7-9 for patient outcomes and fewer than 60% scoring 7-9 for all other outcomes were considered ‘out’. It was proposed to consensus meeting participants that these outcomes should not be re-voted unless otherwise requested. |
| No consensus  n=12 | It was proposed to the consensus meeting participants that the ‘no consensus’ outcomes should be considered in two groups using two pre-determined thresholds:   1. Those outcomes where 70-90% of participants scored 7-9 for patient outcomes and 60-70% of participants scoring 7-9 for all other outcomes (n=11) 2. Those outcomes meeting the above criteria but where there was high disagreement in scoring between stakeholder groups (disagreement of over 40%) (n=1) |
| Wide variation in consensus  N=4 | Outcomes with high disagreement in scoring between stakeholder groups, where overall consensus was achieved but rated outcomes were very highly rated by one group (at least one group scored over 90% but there was disagreement of over 40%). It was proposed that the consensus meeting participants vote yes or no for inclusion or exclusion from the final core set. |

**S11 Table Voting on outcomes during consensus meeting**

| **Outcome level** | **Outcome** | **Score n (%)** | |
| --- | --- | --- | --- |
|  |  | **Yes** | **No** |
| **Patient** |  |  |  |
| *No consensus after R2* |  |  |  |
|  | Overall measure of complications | 14/14 (100%) | 0 |
|  | Time to physical recovery | 4/14 (29%) | 10/14 (71%) |
|  | Post-operative readmission/reoperation | 2/14 (14%) | 12/14 (86%) |
|  | Outcomes post-hospital: Disease progression/treatment failure | 0 | 14/14 (100%) |
|  | *Satisfaction with procedure-specific outcome | 3/13 (23%) | 10/13 (77%) |
|  | Operative progress | 0 | 14/14 (100%) |
|  | Outcomes post-hospital: Subsequent treatment | 2/14 (14%) | 12/14 (86%) |
| *Consensus out after R2 but with wide variation between groups* |  |  |  |
|  | Cardiovascular complications | 0 | 14/14 (100% |
|  | Renal complications | 0 | 14/14 (100% |
|  | *Cerebrovascular complications | 0 | 13/13 (100%) |
|  | Cognitive complications | 0 | 14/14 (100% |
| *Consensus in after R2* |  |  |  |
|  | Procedure-specific injury | 3/14 (21%) | 11/14 (79%) |
|  | Mortality | 3/14 (21%) | 11/14 (79%) |
| **Surgeon** |  |  |  |
| *No consensus after R2* |  |  |  |
|  | Learning curve | 2/14 (14%) | 12/14 (86%) |
|  | *Operative time | 0 | 12/12 (100%) |
|  | Impact on concentration | 0 | 14/14 (100%) |
| *Consensus in after R2* |  |  |  |
|  | Visualisation | 5/14 (36%) | 9/14 (64%) |
|  | Control of instruments | 1/14 (7%) | 13/14 (93%) |
|  | *Surgeon autonomy | 2/13 (15%) | 11/13 (85%) |
| **Organisation** |  |  |  |
| *No consensus after R2* |  |  |  |
|  | Overall Efficiency | 2/14 (14%) | 12/14 (86%) |
|  | Overall economic/cost-effectiveness | 13/14 (93%) | 1/14 (7%) |

*internet connections prevented some participants’ votes from being recorded
